# Formononetin relieves chronic stress-related neointimal hyperplasia in mice by regulating HDAC6

**DOI:** 10.3389/fcvm.2026.1865656

**Published:** 2026-10-07

**Authors:** Shanqian Li, Wanhua Wu, Chaozhi Xu, Yue Huang, Guixian Wu, Hongyu Ding, Zongyi Zhang, Yulin Zhang, Meimei Zheng, Hongxian Wu, Lina Hu

**Affiliations:** 1 The School of Public Health, Guilin Medical University, Guilin, Guangxi, China; 2 Key Cultivation Laboratory of Life Cycle Health Care Research, Guilin, Guangxi, China; 3 Guangxi Key Laboratory of Environmental Exposures and Whole Life Cycle Health, Guilin, Guangxi, China; 4 Department of Clinical Nutrition, Liuzhou Workers’ Hospital, Liuzhou, Guangxi, China; 5 School of Artificial Intelligent Medicine, Guilin Medical University, Guilin, Guangxi, China; 6 Department of Pediatrics, The First Affiliated Hospital of Guangxi Medical University, Nanning, Guangxi, China; 7 Communicable Disease Control Branch, Qingdao Municipal Center for Disease Control and Prevention, Qingdao, Shandong, China; 8 Department of Nutrition, Second People’s Hospital of Ya'an City, Ya'an, Sichuan, China; 9 School of Health and Hygiene, Beihai Health and Wellness Vocational College, Beihai, Guangxi, China; 10 Department of Cardiology, Zhongshan Hospital, Fudan University, Shanghai Institute of Cardiovascular Diseases, Shanghai, China

**Keywords:** autophagy, chronic stress, formononetin, HDAC6, neointimal hyperplasia

## Abstract

**Introduction:**

Chronic stress severely impairs physical and mental health and is a critical risk factor for cardiovascular diseases. Formononetin (FMN) has shown promising cardiovascular protective effects, yet its functional role and molecular mechanism in chronic stress-triggered arterial neointimal hyperplasia and concomitant mood disturbances remain poorly elucidated.

**Methods:**

Network pharmacology was used to predict the underlying mechanisms of FMN against cardiovascular disease-depression comorbidity. A dual mouse model recapitulating chronic stress and carotid artery injury was established for *in vivo* validation. Histological staining, behavioral tests, Western blotting, *β*-galactosidase staining, and *in vitro* VSMC assays were performed to assess the effects of FMN and the HDAC6 inhibitor TubA.

**Results:**

Network pharmacology identified 111 overlapping targets, with functional enrichment and molecular docking revealing that FMN primarily regulates inflammation, apoptosis, and autophagy via binding to core hub proteins including HDAC6, Bcl-2, and AKT1. *In vivo*, chronic stress aggravated arterial intimal hyperplasia and aortic senescence, and induced anxiety- and depression-like behaviors. FMN treatment notably improved emotional disorders, reduced HDAC6 expression, activated autophagy (increased LC3B/A ratio), inhibited apoptosis (elevated Bcl-2/Bax ratio), attenuated NLRP3 and IL-6 expression, and alleviated intimal thickening. The HDAC6 inhibitor TubA recapitulated the vascular protective effects of FMN.

**Discussion:**

FMN attenuates chronic stress-induced vascular injury and emotional dysfunction via suppressing HDAC6 signaling to restore autophagy, inhibit inflammation, and reduce apoptosis. This study unveils the dual cardio-neuroprotective capacities of FMN and provides novel mechanistic insights for the treatment of chronic stress-related cardiocerebral comorbidities.

## Graphical Abstract

**Figure:**
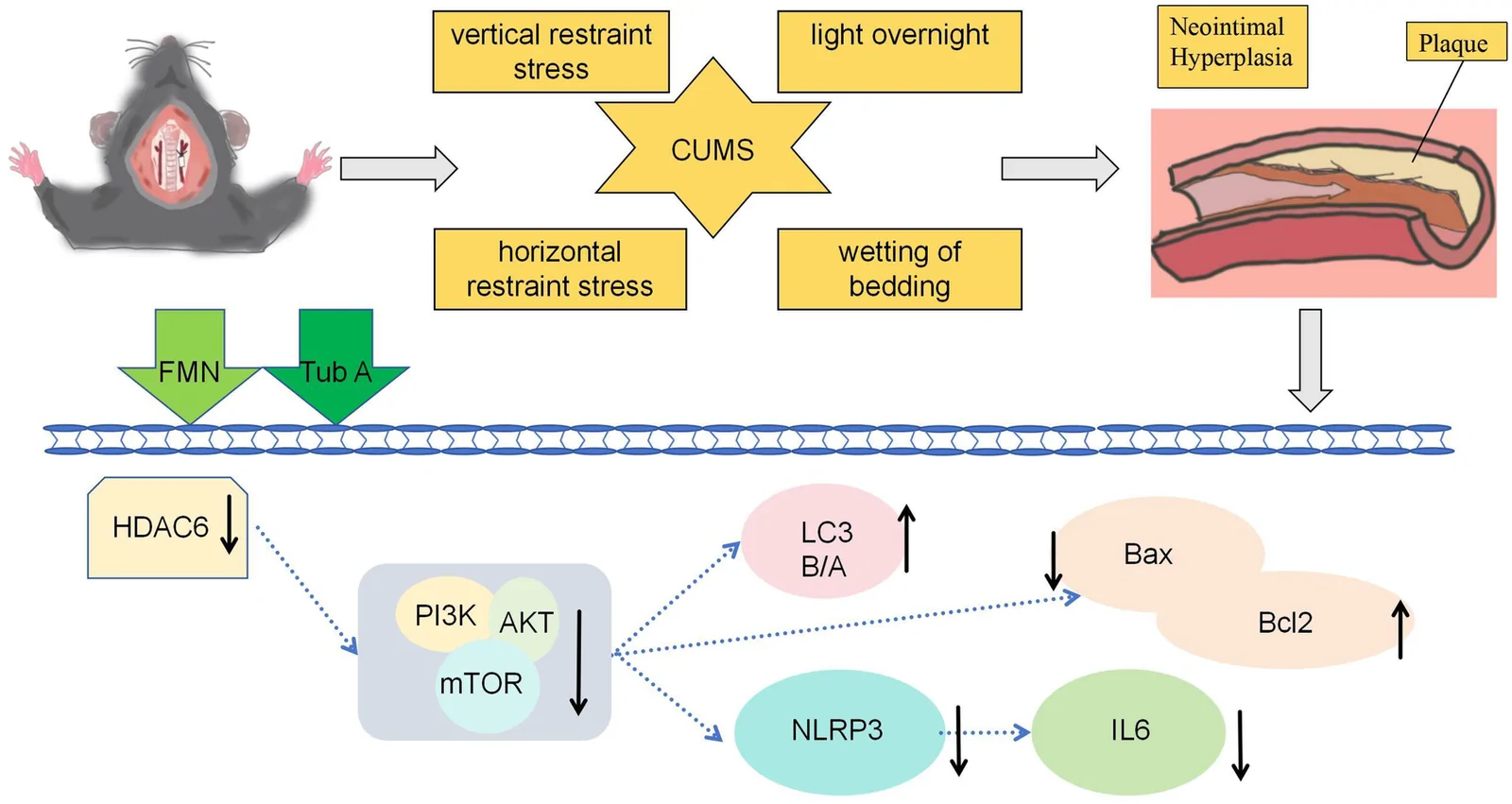

## 1 Introduction

In recent years, the prevalence of anxiety and depression has risen sharply among populations exposed to chronic high-pressure environments, driven by factors including life changes(e.g., marital discord, natural disasters) and unfavorable socioeconomic conditions(e.g., low income, high crime rates) (1). When individuals are persistently subjected to unpredictable, uncontrollable social stressors, sustained physiological and behavioral adaptations emerge, leading to cumulative bodily damage on underlying physiological functions, stress-induced pathological effects are more likely to develop (2). Chronic stress(CS) is a non-specific pathophysiological response caused by the alteration of the homeostasis of the organism under the long-term stimulation of various adverse factors inside and outside the body(internal and external environment, somatic, psychological, and social factors) (3). Excessive or prolonged activation of the hypothalamic-pituitary-adrenal(HPA) axis under conditions of overwhelming stress load or chronic recurrent stress exposure may induce dysregulation across multiple physiological systems (4), including immune responses, neurochemical homeostasis, and endocrine function (5). It is noteworthy that chronic stress not only adversely affects mental health, increasing the risk of anxiety and depression, but also elevates the risk of various diseases including cardiovascular disease(CVD) and chronic inflammation (6, 7). Among the 20.5 million CVD-related deaths recorded in 2021, 80% occurred in low- and middle-income countries (8). Global research on CVD burden and risk factors indicates that the total number of CVD cases currently exceeds 587 million, with the overall prevalence continuing to rise annually (9). Cardiovascular disease remains the leading cause of mortality worldwide (10). Therefore, comprehensive research on chronic stress-mediated multisystemic and multidisorder effects is urgently needed. In this study, we will focus specifically on the impact of chronic stress on cardiovascular diseases.

Neointimal hyperplasia refers to the persistent vascular wall inflammation, abnormal proliferation and migration of vascular smooth muscle cells(VSMCs) following vascular injury or stimulation, serving as a common pathological basis for various cardiovascular diseases including postoperative restenosis. Neointimal hyperplasia predominantly develops at sites of vascular endothelial injury, such as in vascular segments following interventional procedures (11). As one of the most commonly used interventional techniques for arterial reconstruction in clinical practice, endovascular intervention has been widely applied in treating patients with coronary artery disease, carotid artery stenosis, and arteriosclerosis obliterans. According to statistics, the incidence of restenosis after carotid endarterectomy(CEA) or carotid artery stenting(CAS) ranges from 3% to 30%, typically occurring within 6 months post-percutaneous transluminal angioplasty(PTA) (12).

Neointimal hyperplasia represents the core pathological mechanism underlying restenosis following vascular interventional procedures(such as stent implantation), which essentially constitutes a pathological structural remodeling resulting from dysregulated vascular repair responses post-injury. Our preliminary research findings demonstrate that modulation of histone deacetylase 6(HDAC6) activation can mediate the control of injury-associated vascular repair by regulating vascular smooth muscle cell(VSMC) proliferation and migration (13). HDAC6, a member of the histone deacetylase family, has a rich set of functions and is required to regulate the proliferation and migration of various types of cancer cells. Under normal conditions, HDAC6 is localized in the cytoplasm and regulates non-histone deacetylation modifications or plays non-enzymatic roles; in some diseases, such as neurodegenerative diseases, HDAC6 enters the nucleus and regulates histone deacetylation modifications, which can inhibit cell differentiation, proliferation, etc., by participating in transcriptional repression. Meanwhile, HDAC6 also regulates protein homeostasis and can usually interact with proteasome degradation-related proteins, thereby modulating responses such as autophagy and heat shock response (14).In addition, the ubiquitin-binding structural domain of HDAC6 is critical for nucleotide-binding oligomerization domain-like receptor protein 3(NLRP3) (15).

Autophagy is an evolutionarily conserved catabolic process that enables cells to remove unwanted proteins and damaged organelles. Normal autophagic activity may be associated with physiological stress response mechanisms, and autophagy defects are induced in subjects exposed to excessive or chronic stress, especially in patients suffering from neuropsychiatric disorders such as major depressive disorder (16). Apoptosis is the genetically controlled autonomous and orderly death of cells, unlike passive cell necrosis, which participates in developmental, immune, and homeostatic regulation by removing damaged, senescent, or redundant cells (17). It blocked PI3 K/Akt/mTOR signaling to activate autophagy and inhibit the onset of apoptosis (18). The interplay between apoptosis and autophagy is critical for the regulation of intracellular homeostasis, and disruption of this balance leads to pathophysiological consequences, including cancer and autoimmune diseases. Induction of autophagic or apoptotic programs are two common responses to cellular stress. Abnormal autophagy or apoptotic in vascular smooth muscle cells is associated with the progression of neointimal hyperplasia-induced vascular remodeling disease.

New evidence suggests that HDAC6 is one of the potential targets of flavonoids (19), formononetin (FMN), a natural isoflavonoid mainly found in *Huang Qi*, *Suberect Spatholobus Stem*, and *Trifolium pratense L*, is a typical phytoestrogen with a wide range of potential pharmacological and biological effects, including anti-inflammatory, antioxidant, antidepressant, and inhibition of tumor cell proliferation and other effects (20). Signaling pathways such as HDAC class and p38 mitogen-activated protein kinase(MAPK) have been reported to be involved in the treatment of Alzheimer's disease(AD) with formononetin (21). This led us to hypothesize whether FMN could regulate the proliferation and migration of vascular smooth muscle cells through the HDAC6 pathway and play an ameliorative role in chronic stress-induced postoperative restenosis.

## 2 Materials and methods

### 2.1 Network pharmacology

Potential molecular targets of FMN were retrieved from SwissTargetPrediction and PharmMapper databases. Genes associated with intimal hyperplasia-related CVDs and depression were collected from GeneCards and DisGeNET. Venny software was used to screen overlapping targets between FMN and the two disease phenotypes. The STRING platform was employed to construct protein-protein interaction (PPI) networks, which were visualized and subjected to topological analysis via Cytoscape to identify core hub genes. Gene Ontology (GO) and Kyoto Encyclopedia of Genes and Genomes (KEGG) enrichment analyses of intersecting targets were performed on the DAVID platform with a significance cutoff of *P* < 0.05. Finally, AutoDock Vina was utilized for molecular docking simulations to quantify binding affinity between FMN and core hub proteins.

### 2.2 Experimental animals

All animal experimental protocols were reviewed and approved by the Laboratory Animal Ethics Committee of Guilin Medical University (Approval No. GLMC202303204), and all animal handling complied with the Guide for the Care and Use of Laboratory Animals. Specific pathogen-free (SPF) male C57BL/6J mice aged 8–10 weeks with body weights of 16–18 g were purchased from Hunan Slake Jinda Laboratory Animal Co., Ltd. [License No. SCXK(Hunan) 2021-0002]. Mice were housed in an SPF animal facility under controlled conditions (temperature: 22 ± 2 ℃; relative humidity: 50 ± 10%; 12 h light/dark cycle) with *ad libitum* access to standard chow and sterile water. All animals underwent a one-week acclimation period prior to experimental manipulations.

### 2.3 Drug administration regimen

Mice were randomly assigned to six experimental groups (*n* = 10 per group): non-stress group, chronic stress group, vehicle intragastric (i.g.) group, FMN i.g. group (10 mg/kg) (22), vehicle intraperitoneal (i.p.) group, and TubA i.p. group (10 mg/kg) (13). Intragastric administration was initiated on Day 1 of the experimental timeline. Tubastatin A (TubA), a selective HDAC6 inhibitor, was delivered via intraperitoneal injection starting three days before carotid injury surgery, consistent with previously established protocols. FMN (purity=99.67%) and TubA (purity = 98.37%) were purchased from MedChemExpress (MCE, USA). Body weight was recorded daily at 8:00 a.m. for dose adjustment, and chronic stress exposure commenced one hour after daily drug delivery (9:00 a.m.).

### 2.4 Establishment of chronic stress and carotid injury dual mouse model

#### 2.4.1 Animal grouping

All groups except the non-stress group received four weeks of chronic unpredictable mild stress (CUMS) combined with right carotid artery cuff injury to construct the composite disease model.

#### 2.4.2 Modeling of chronic stress

After a 1 week acclimatization period, mice in the stress groups were randomly exposed to two different stimuli at fixed times (9:00 a.m. to 1:00 p.m.) daily for 4 weeks, while non-stressed mice were allowed free movement without any stimulation. The stress protocols are illustrated in Figure 1A, and the restraint stress procedure is shown in Figure 1B.

**Figure 1:**
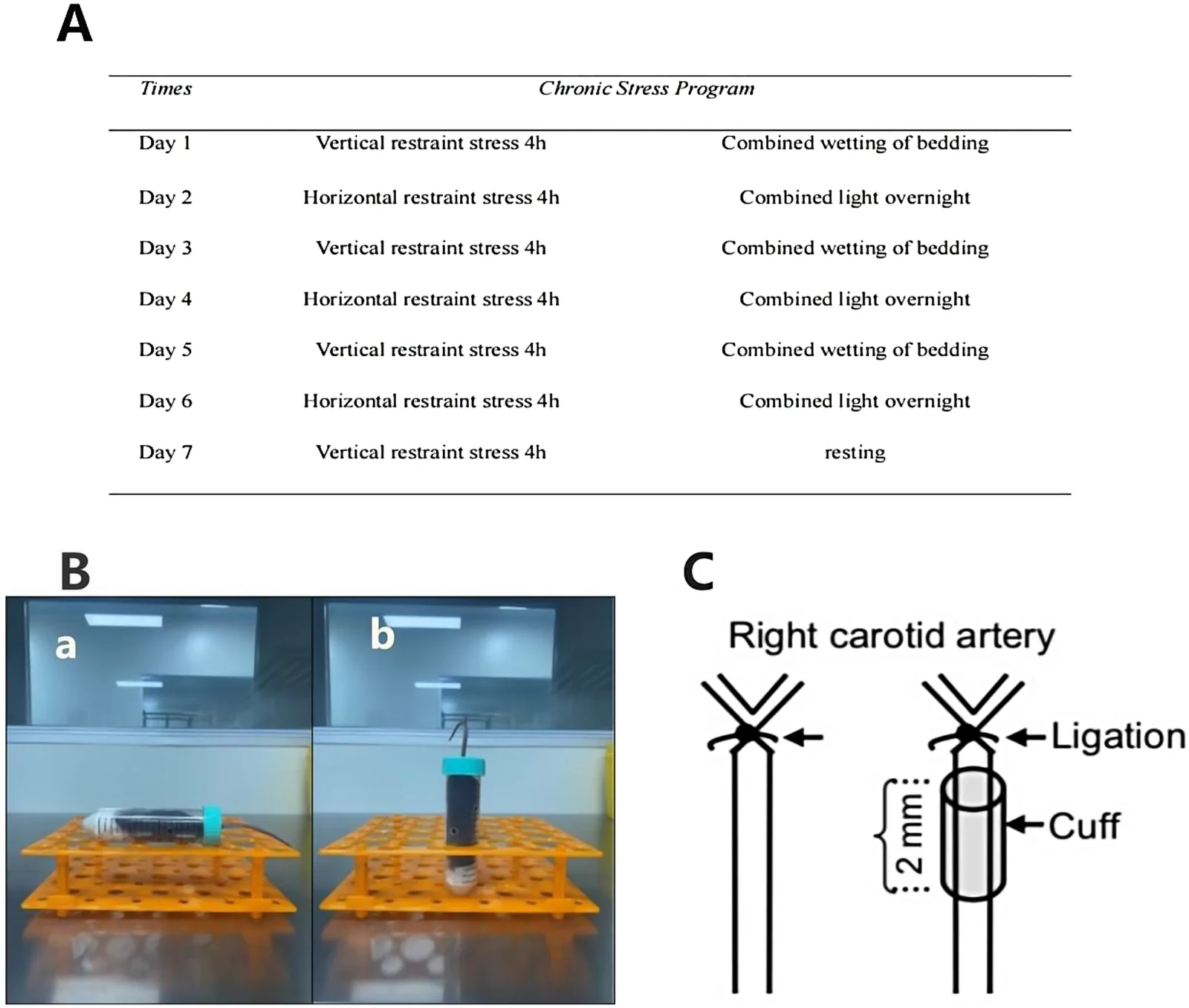
CUMS stress protocols and schematic diagram of carotid cuff injury. **(A)** Weekly schedule of chronic unpredictable mild stress stimulation; **(B)** Representative images of vertical restraint **(b)** and horizontal restraint **(a)** mouse models; **(C)** Schematic illustration of right carotid artery ligation and polyethylene cuff placement, with marked distance between ligation and cuff (2 mm).

#### 2.4.3 Right carotid artery injury

Each group of mice underwent right carotid artery injury surgery on day 14 at the beginning of the experimental procedure to create a dual model of chronic stress and carotid artery injury (23).

On Day 14 of the experimental timeline, all mice underwent right common carotid artery injury under pentobarbital sodium anesthesia (Figure 2). After complete loss of pain reflexes, mice were fixed on a surgical board, and the cervical region was disinfected with 75% ethanol. A small cervical incision was made to isolate the right common carotid artery, which was ligated with surgical silk proximal to the carotid bifurcation. A non-occlusive polyethylene cuff (length = 2 mm, inner diamete*r* = 0.580 mm, outer diamete*r* = 0.965 mm) was placed approximately 2 mm distal to the ligation site and secured circumferentially with sutures. The surgical incision was closed with absorbable sutures after confirmation of hemostasis.

**Figure 2:**
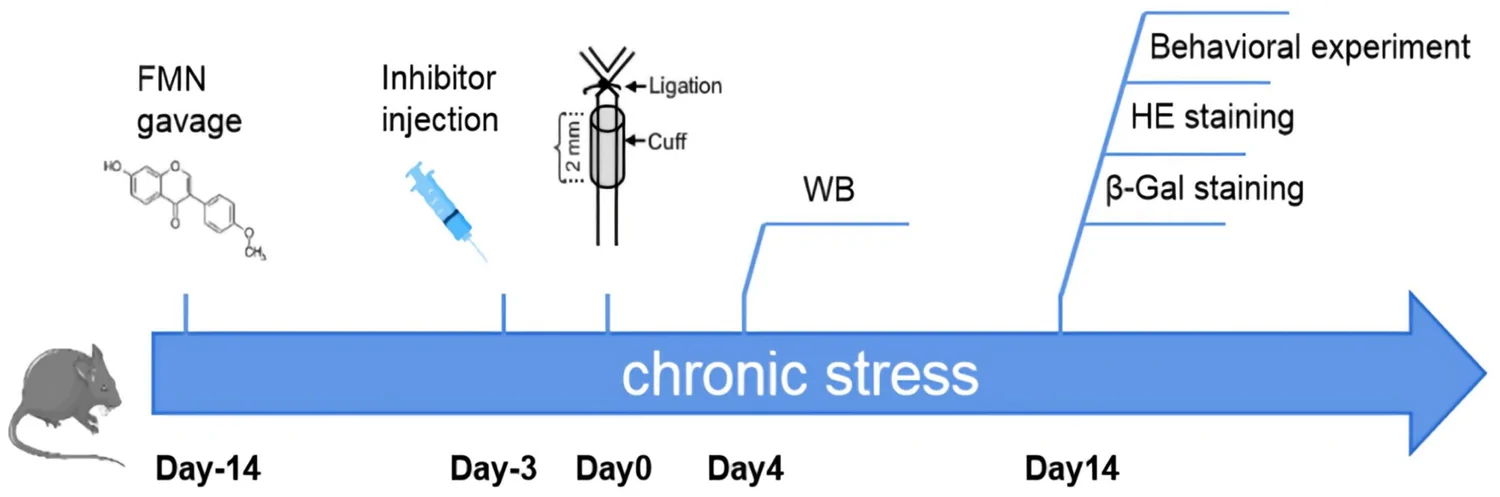
Overall experimental timeline. Timeline illustrating FMN intragastric pre-administration (Day −14), TubA intraperitoneal pre-injection (Day −3), carotid cuff injury surgery (Day 0), WB tissue collection (Day 4 post-operation), and terminal detection including behavioral tests, H&E histology and aortic *β*-Gal senescence staining (Day 14 post-operation), all under sustained chronic stress exposure.

### 2.5 Behavioral assessments for anxiety- and depression-like phenotypes

#### 2.5.1 Sucrose preference test (SPT)

Mice were single-housed and acclimated to two bottles of 1% sucrose solution for 24 h (24). Subsequently, one sucrose bottle and one water bottle were provided, with bottle positions swapped every 12 h to eliminate side bias. After 12 h of food and water deprivation, sucrose consumption and water intake were recorded over a 24 h testing period, with bottle positions exchanged every 12 h. Sucrose preference (%) = sucrose intake/(sucrose intake + water intake) × 100, a readout of anhedonia.

#### 2.5.2 Forced swim test (FST)

Mice were placed in a cylindrical tank (diamete*r* = 20 cm, height = 30 cm) filled with water (temperature = 23 ± 1 °C, depth = 16 cm) (25). After a 2-min habituation phase, immobility duration during the subsequent 4 min was quantified; prolonged immobility indicates behavioral despair, a core depressive-like trait.

#### 2.5.3 Open field test (OFT)

Each mouse was placed in the center of a 50 cm × 50 cm × 40 cm open field arena and allowed to freely explore for 10 min (26). Locomotor trajectories were tracked using Super Maze software, and the percentage of time spent within the central 25 cm × 25 cm zone was calculated as an index of anxiety-like behavior.

### 2.6 Euthanasia and sampling

Mice were fasted for 12 h prior to surgery. At the end of the experimental procedures, all mice were deeply anesthetized with sodium pentobarbital (150 mg/kg, i.p.). After loss of the pedal withdrawal reflex, animals were euthanized by exsanguination via cardiac puncture. Death was confirmed by respiratory arrest and absence of heartbeat. All efforts were made to minimize suffering, and the euthanasia method adhered to the AVMA Guidelines for the Euthanasia of Animals.

Blood samples were collected in 1.5 mL centrifuge tubes, allowed to clot at room temperature for 2 h, and centrifuged at 4℃ and 4,000 rpm for 15 min. The serum was separated and stored at −80℃.

Following blood collection, mice were transcardially perfused with physiological saline via the left ventricle using a 20 mL syringe to remove residual blood. For the 4-day postoperative group, the right common carotid artery was harvested, immediately frozen in liquid nitrogen, and stored at −80℃ for Western blot analysis. For the 14-day postoperative group, the right common carotid artery and the aorta were placed in tissue fixative for subsequent histopathological staining. In each group (*n* = 10), five mice were used for histological and morphometric analyses, and the remaining five for biochemical assays (e.g., Western blot).

### 2.7 Preparation of frozen sections of right carotid artery

Carotid artery tissues were fixed in 4% paraformaldehyde at 4°C overnight, rinsed with 1× PBS, and dehydrated in sucrose solution until the tissue sank. Samples were then horizontally placed in aluminum foil embedding cassettes, filled with OCT compound without air bubbles, rapidly frozen in liquid nitrogen, and stored at −20°C.

Prior to sectioning, the cryostat was pre-cooled. Embedded blocks were fixed onto the specimen holder with the vessel long axis perpendicular to the blade. Serial cross-sections (5–6 μm thick) were cut approximately 2 mm from the ligation site and stored at −20°C for subsequent use.

### 2.8 Histomorphometric quantification

Carotid artery tissues were fixed in formalin, embedded in paraffin, and cut into continuous 5 μm-thick sections. Sections were stained with hematoxylin and eosin (H&E) and elastic Van Gieson (EVG) staining to visualize vascular wall architecture. Bright-field microscopy was used to capture histological images, and Image J software was applied to quantify vascular compartment areas as follows:

Media area = external elastic lamina (EEL) area—internal elastic lamina (IEL) area. Neointimal area = internal elastic lamina (IEL) area—lumen area. Six cross-sections (two proximal, two medial, two distal per vessel) were quantified and averaged for each sample to eliminate regional variation.

### 2.9 Western blot assay

Frozen carotid artery tissues were ground in liquid nitrogen and lysed in RIPA buffer to extract total protein, whose concentration was determined via the BCA assay. Equal amounts of denatured protein were separated by SDS-PAGE and transferred to PVDF membranes. After blocking, membranes were incubated with primary antibodies overnight at 4 °C at a dilution of 1:1000: *p*-p38, p38, *p*-PI3 K, PI3 K, *p*-AKT, AKT, *p*-mTOR, LC3B/A (Cell Signaling Technology, USA); Bax, Bcl-2 (Abcam, USA); *p*-HDAC6 (Invitrogen, USA); HDAC6, *β*-actin (Wuhan Sanying Biotechnology, China). Membranes were washed three times with PBS-T and incubated with fluorescent secondary antibodies (1:10,000, Thermo Fisher, USA) for 2 h at room temperature. Protein bands were visualized using a fully automated multi-channel chemiluminescence imaging system (BIO-RAD, USA), and band gray values were quantified for statistical comparison.

### 2.10 Senescence-associated *β*-galactosidase (*β*-Gal) staining

Fresh aortic segments were placed in 2 mL centrifuge tubes and fixed with *β*-Gal fixative for 15 min at 25 °C, followed by three PBS washes (2 min each). Tissues were incubated with *β*-Gal staining solution at 37 °C in a humidified incubator until blue senescence-positive staining emerged. Stained aortas were imaged under light microscopy to compare the extent of vascular senescence across groups.

### 2.11 *In vitro* vascular smooth muscle cell (VSMC) assays

Primary mouse aortic VSMCs were purchased from Shanghai Fuhang Biotechnology Co., Ltd. (Cat. No. FH1289). Cells were cultured in complete DMEM medium supplemented with 10% fetal bovine serum and 1% penicillin-streptomycin, maintained at 37 °C with 5% CO₂ in a humidified incubator.

#### 2.11.1 CCK-8 proliferation assay

VSMCs were serum-starved overnight, digested with trypsin, and seeded into 96-well plates at a density of 1 × 10^3^ cells per well. Cells were treated with vehicle or 10 μM FMN for 24 h and 48 h. Additional experimental groups included basal DMEM, DMEM supplemented with 1% serum harvested from CUMS-exposed mice, and 1% CUMS mouse serum + 10 μM FMN. Each group contained three technical replicates. After treatment, 10 μL CCK-8 solution was added to each well, followed by 1.5 h incubation in the dark. Optical density (OD) at 450 nm was measured via a microplate reader to quantify cell proliferative activity. All cellular experiments were repeated in biological triplicate.

#### 2.11.2 Scratch wound healing migration assay

Horizontal reference lines were pre-marked on the bottom of six-well plates at 1 cm intervals. Confluent VSMC monolayers were scratched vertically with sterile pipette tips, and detached cells were washed away with PBS. Microscopic images of wound areas were captured at 0, 12, 24 and 48 h post-scratching. The migration percentage was calculated as: (initial scratch area at 0 h—residual scratch area at indicated timepoint)/initial scratch area at 0 h × 100%.

#### 2.11.3 Flow cytometry analysis of cell apoptosis

VSMCs from all experimental groups were harvested and washed with PBS, then stained with the Annexin V-FITC/PI apoptosis detection kit in the dark following manufacturer protocols. Cellular apoptosis rates were analyzed by flow cytometry with FlowJo software, with three independent biological replicates performed.

### 2.12 Statistical methods

All quantitative data were analyzed using GraphPad Prism 8 and presented as mean ± standard deviation (mean ± SD). Two-group comparisons were conducted via unpaired Student's t-test, while multiple intergroup comparisons were performed using one-way analysis of variance (ANOVA) followed by Tukey's *post-hoc* test. *p* < 0.05 was defined as the threshold for statistically significant differences.

## 3 Results

### 3.1 Network pharmacology predicts FMN targets for CVD-depression comorbidity

A total of 131 FMN-related targets were retrieved from the TCMSP, SwissTargetPrediction, PharmMapper, and CTD databases. After removing duplicates, 111 unique targets were retained as potential FMN targets. Meanwhile, targets associated with intimal hyperplasia-related cardiovascular disease (CVD) and depression were collected from GeneCards, DisGeNET, OMIM, TTD, and CTD databases. Following duplicate removal, 33,436 CVD-related and 17,620 depression-related targets were obtained. Intersection of the merged CVD–depression target set with the 111 FMN targets yielded 111 common targets, which were considered candidate targets mediating the therapeutic effects of FMN on CVD–depression comorbidity (Figure 3A).

**Figure 3:**
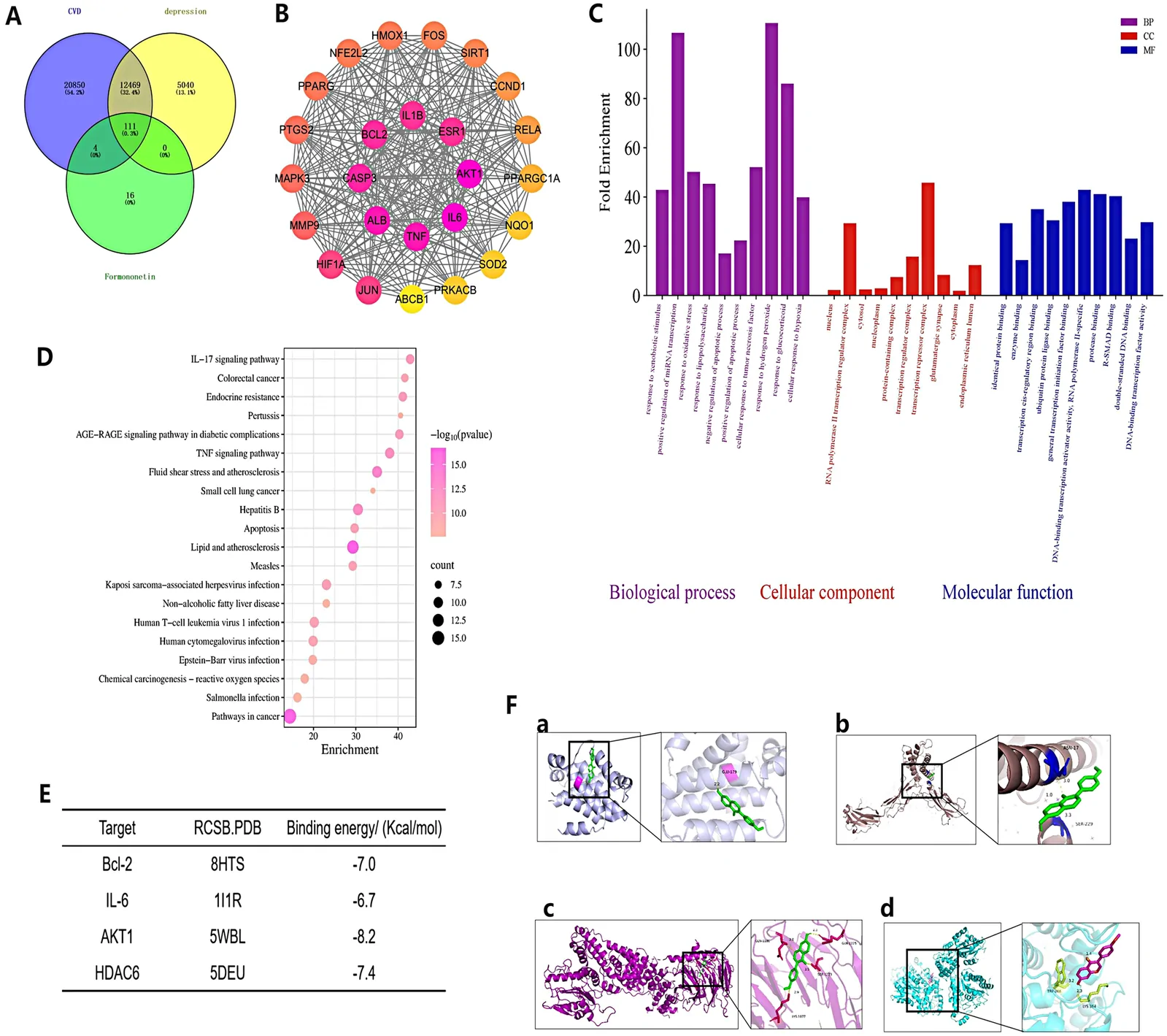
Network pharmacology analytical results. **(A)** Venn diagram displaying overlapping targets of FMN, CVD and depression; **(B)** Protein-protein interaction (PPI) network of core intersecting hub genes, with node size proportional to interaction degree; **(C)** GO enrichment bar chart grouped by biological process (BP), cellular component (CC) and molecular function (MF); **(D)** KEGG pathway enrichment scatter plot; **(E)** Table summarizing binding energy values of FMN with HDAC6, Bcl-2, AKT1 and IL-6; **(F)** 3D molecular docking conformations of FMN bound to Bcl-2 **(a)**, IL-6 **(b)**, AKT1 **(c)** and HDAC6 **(d).**

These 111 overlapping targets were imported into the STRING online platform for protein–protein interaction (PPI) network analysis. The obtained interaction data were subsequently imported into Cytoscape 3.7.1 for topological analysis. The Network Analyzer tool was employed to calculate the topological parameters of each node, and three core hub genes were ultimately identified as the core targets of FMN in the treatment of CVD–depression: Bcl-2, IL-6, AKT1 (Figure 3B).

To further clarify the biological functions of FMN-related targets in the context of CVD–depression, Gene Ontology (GO) and Kyoto Encyclopedia of Genes and Genomes (KEGG) pathway enrichment analyses were conducted on the 111 overlapping targets (Figures 3C,D).

Based on the network pharmacology results, the three hub proteins (Bcl-2, IL-6, and AKT1) were selected for molecular docking analysis, where FMN was docked with each of these three key proteins. It is noteworthy that, although HDAC6 was not ranked among the core targets by topological analysis of the PPI network, a previous network pharmacology study has demonstrated that the biological processes regulated by FMN are significantly enriched in HDAC-related signaling pathways, suggesting a potential functional association between FMN and HDAC family members (21). In the context of vascular injury repair, the PI3K-Akt/*p*-HDAC6 signaling axis has been firmly established as a critical regulator of VSMC proliferation and neointimal hyperplasia (13). Based on the above literature evidence and our pathway enrichment results, we included HDAC6 as a complementary target for molecular docking to explore whether FMN directly interacts with this upstream regulator, thereby bridging the network pharmacology predictions with the subsequent mechanistic validation.

Given that it can regulate multiple predicted targets such as AKT1 and IL-6, it was included for validation as an upstream regulatory factor. Notably, HDAC6, Bcl-2, and AKT1 exhibited strong binding affinity with FMN (binding energy < −7.0 kcal/mol) (Figure 3E). Schematic diagrams of the molecular docking results are presented in Figure 3F.

### 3.2 FMN mitigates chronic stress-induced neointimal hyperplasia and mood disorders

#### 3.2.1 Chronic stress exacerbates vascular intimal thickening and induces anxiety/depression-like behaviors

To visually assess the degree of carotid intima-media thickening, frozen sections of the right carotid artery from injured mice were subjected to H&E and EVG staining. The extent of neointimal hyperplasia was evaluated by calculating the ratio of neointimal area to medial area in H&E-stained sections (27).

Histological analysis was performed on postoperative day 14. Compared with the non-stressed group, the right carotid artery of chronically stressed mice exhibited marked intima-media thickening and severe luminal narrowing (Figure 4A). Quantitative analysis revealed that both the neointimal area (Figure 4B) and the neointima-to-media ratio (Figure 4C) were significantly increased in the stress group (both *p* < 0.05). These findings confirmed the successful establishment of the chronic stress-induced postoperative restenosis model and indicated that chronic stress exacerbates injury-induced neointimal hyperplasia.

**Figure 4:**
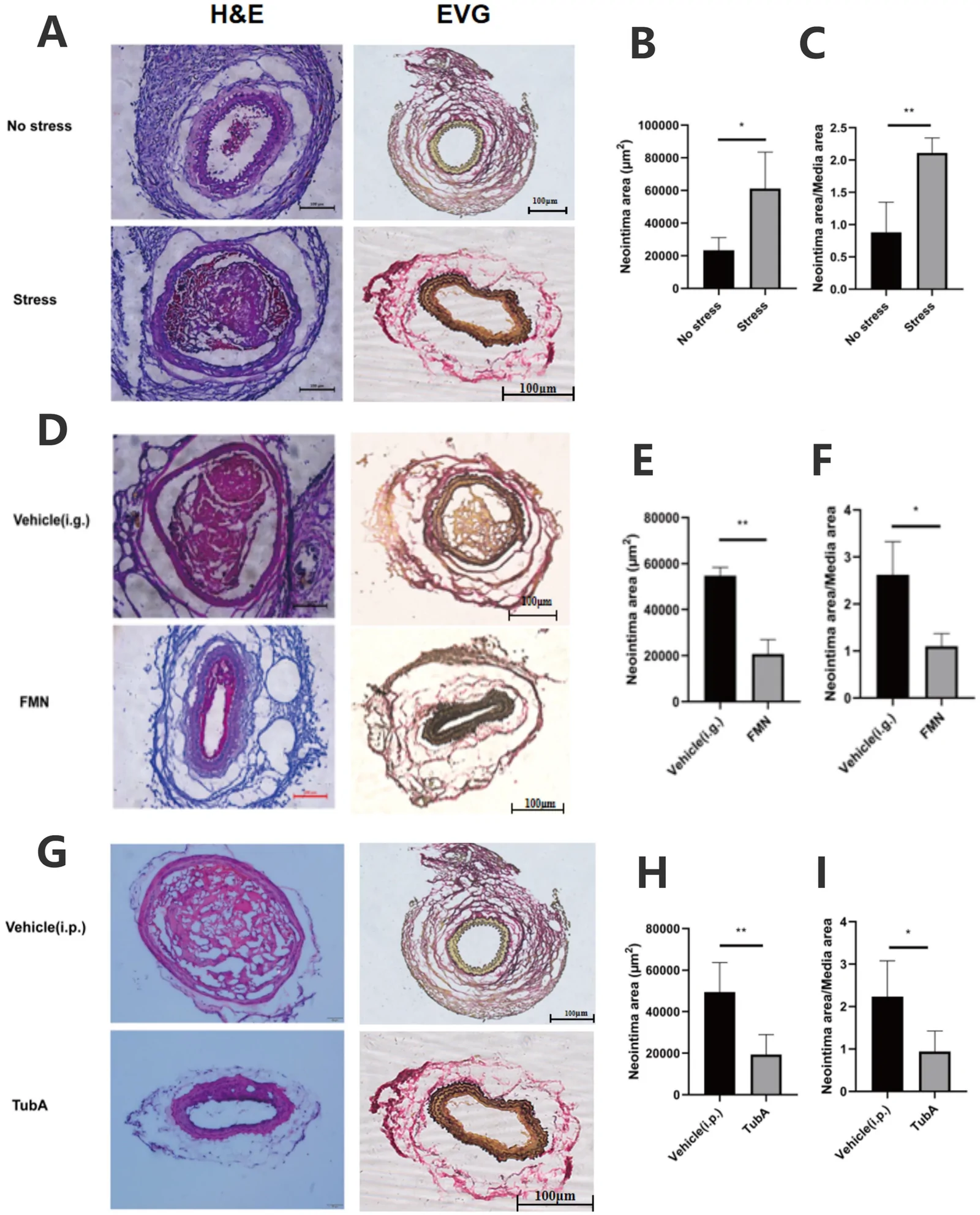
H&E and EVG staining and quantitative morphometry of carotid neointimal hyperplasia at post-operative Day 14 (*n* = 5). **(A–C)** Representative vascular cross-sections and statistical quantification of neointimal area and neointima/media ratio in non-stress vs. chronic stress groups; **(D–F)** Histological images and quantitative analysis of vehicle i.g. vs. FMN groups; **(G–I)** Histological images and quantitative analysis of vehicle i.p. vs. TubA groups. *p < 0.05, **p < 0.01.

In parallel, depressive- and anxiety-like behaviors were evaluated via sucrose preference test (SPT), forced swimming test (FST), and open field test (OFT). Compared with non-stressed mice, the stress group showed a significantly lower sucrose preference rate (*p* < 0.05, Figure 5A), a significantly prolonged immobility time in the FST (*p* < 0.05, Figure 5B), and a significantly reduced percentage of time spent in the central zone of the OFT (*p* < 0.05, Figures 5C,D). These results indicate that chronic stress not only promotes vascular neointimal hyperplasia but also induces depression- and anxiety-like behaviors in mice.

**Figure 5:**
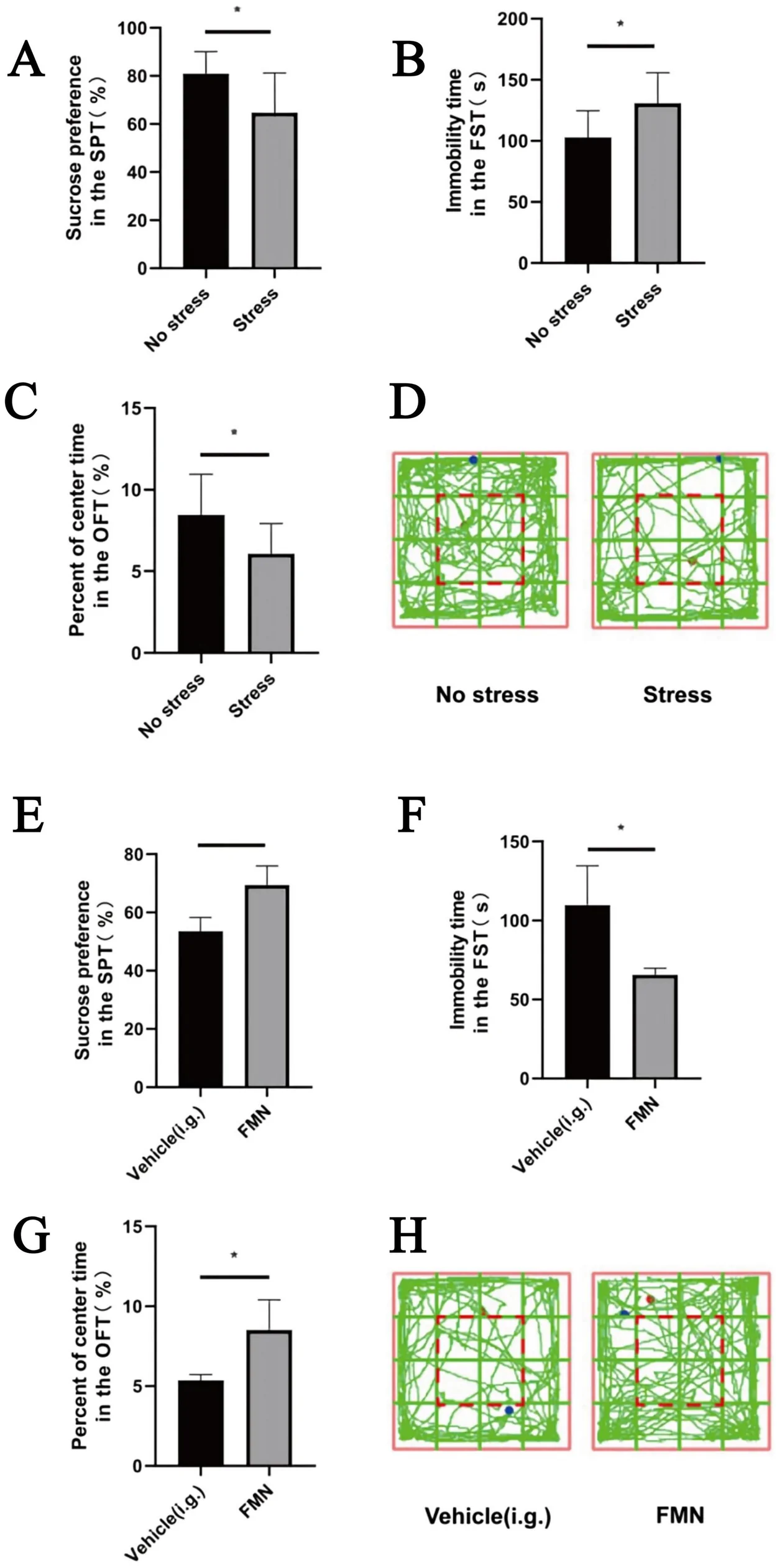
Behavioral assessments of anxiety and depression-like phenotypes. **(A,E)** Sucrose preference rate from SPT; **(B,F)** Immobility duration recorded in FST; **(C,G)** Percentage of time spent in the central zone during OFT; **(D,H)** Representative locomotor trajectory heatmaps from OFT. SPT, sucrose preference test, FST, forced swim test; OFT, open field test, *n* = 5, *p < 0.05.

#### 3.2.2 FMN administration rescues vascular lesions and ameliorates stress-triggered mood dysfunction

The above results confirm that chronic stress causes both physiological and psychological damage in mice after carotid artery injury surgery, but it remains to be investigated whether formononetin can effectively alleviate these pathological changes under chronic stress. Therefore, we gave FMN gavage as well as equal doses of solvent gavage to chronic stress and right carotid artery injury surgical double model mice, respectively, and performed relevant behavioral tests and H&E staining and EVG staining on frozen sections of right carotid artery to observe the emotional changes and vascular remodeling of mice.

As shown by the H&E staining and EVG staining results of the carotid arteries on postoperative day 14 under sustained chronic stress, FMN administration alleviated the thickening of the intima-media and stenosis of the lumen of the right carotid artery in mice compared with the solvent gavage control group (Figure 4D). Quantitative results showed that FMN administration significantly reduced the neointimal area(*p* < 0.01) (Figure 4E) and lowered the ratio of neointimal area to mesointimal area(*p* < 0.05) (Figure 4F), and the difference was statistically significant. This suggests that FMN treatment effectively alleviated the development of chronic stress-induced postoperative restenosis.

Behavioral results showed that sucrose preference rate in the FMN group rebounded(*p* < 0.05) compared to the solvent gavage control group (Figure 5E), immobility time in forced swimming was significantly shorter(*p* < 0.05) (Figure 5F), and the percentage of time spent in the central area of the open field experiment was significantly higher(*p* < 0.05) (Figures 5G,H), all of which were statistically significant differences. These results suggest that FMN administration simultaneously improved depression and anxiety-like behaviors in mice caused by chronic stress.

### 3.3 FMN attenuates stress-related neointimal hyperplasia via HDAC6 suppression

#### 3.3.1 Chronic stress upregulates HDAC6 activation, which is reversed by FMN

In our previous study, we demonstrated that carotid artery vascular injury in mice increased the phosphorylation levels of p38 mitogen-activated protein kinase, Akt, and the cell migration regulator HDAC6 in ligated arteries to regulate chronic stress-associated neointimal hyperplasia (13), suggesting that the HDAC6 pathway plays an important role in the stress-associated vascular remodeling after injury. To further investigate the specific mechanism of FMN to ameliorate chronic stress-induced postoperative restenosis, the present study examined the effect of FMN on the expression of HDAC6-related proteins in the right carotid artery tissues of mice in dual-model mice by using western blot(WB).

WB results showed (Figure 6A) that *p*-HDAC6/HDAC6 levels(*p* < 0.05) (Figure 6B) in the right carotid arteries of the FMN group of mice, were also significantly reduced compared with solvent-gavaged control mice on postoperative day 4 in the sustained chronic stress condition, and the differences were all statistically significant. It is suggested that FMN may play a role in attenuating the pathologic hyperplasia of the injured endothelium by inhibiting the HDAC6 pathway.

**Figure 6:**
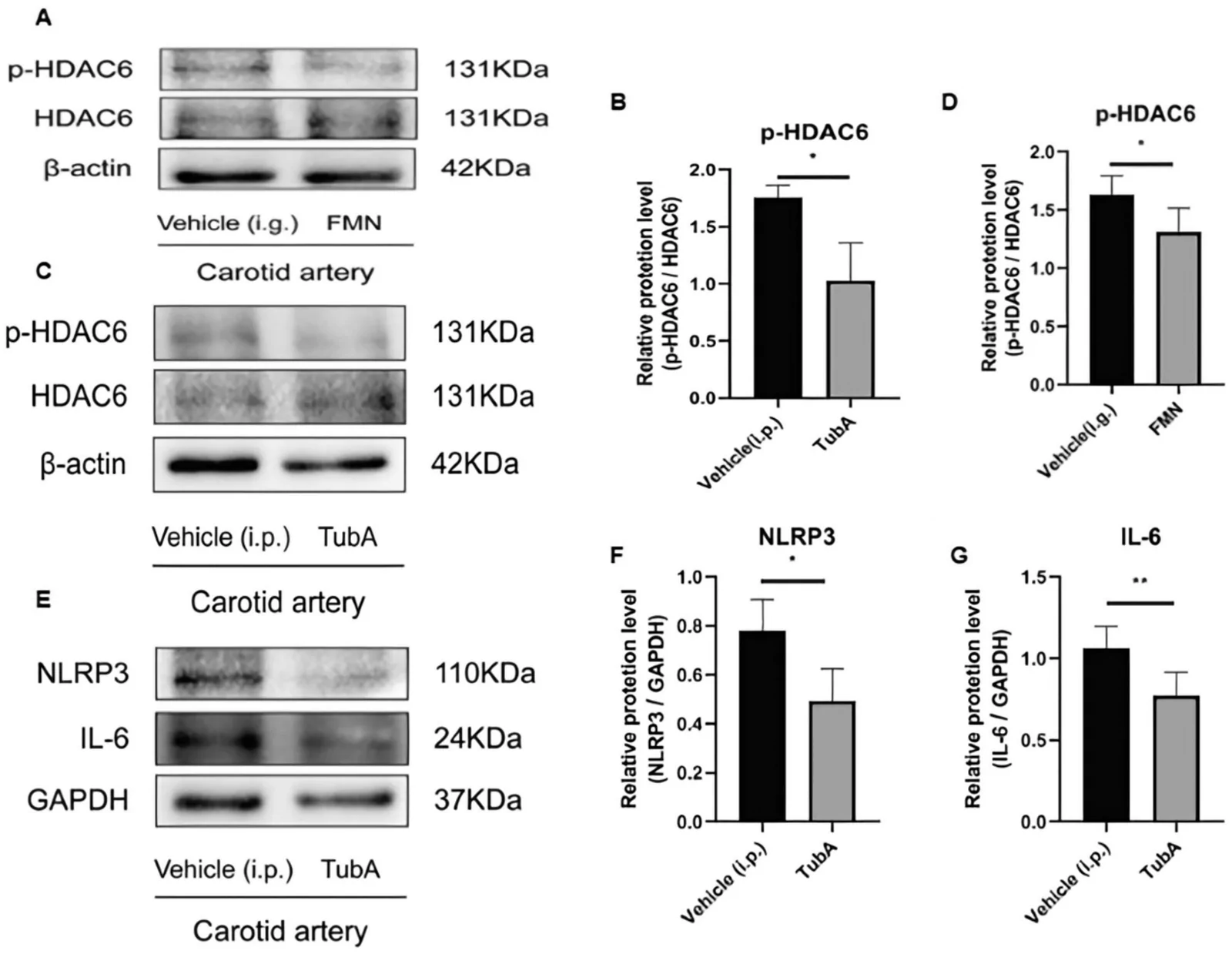
Western blot detection of HDAC6, NLRP3 and IL-6 protein expression in carotid tissues at post-operative Day 4 (*n* = 5). **(A,B)** *p*-HDAC6/HDAC6 band profiles and relative gray quantification of vehicle i.g. vs. FMN groups; **(C,D)** *p*-HDAC6/HDAC6 band profiles and relative gray quantification of vehicle i.p. vs. TubA groups; **(E–G)** Immunoblot bands and quantitative analysis of NLRP3 and IL-6 in vehicle i.p. vs. TubA groups. *p < 0.05, **p < 0.01.

#### 3.3.2 HDAC6 inhibition recapitulates FMN-mediated vascular protection

A large number of previous studies have elucidated the relationship of HDAC6 in the mechanism of restenosis (13), to further verify whether inhibition of the HDAC6 pathway can attenuate the pathological thickening of the carotid artery intima associated with chronic stress-induced injury, the present study continued to treat mice in the dual model of chronic stress and carotid artery injury by intraperitoneal injection of the HDAC6 inhibitor, TubA, to observe the pathological thickening of the neointima and changes in the expression levels of the above proteins in the injured arterial tissues.

As shown in Figure 3, on postoperative day 14 under sustained chronic stress, TubA administration was equally effective in alleviating intimal thickening and luminal stenosis of the right carotid artery in mice compared with solvent intraperitoneal injection controls(Figure 4G). Quantitative results showed that TubA administration significantly reduced the neointimal area(*p* < 0.01)(Figure 4H) and lowered the ratio of neointimal area to intima-media area(*p* < 0.05)(Figure 4I), and the differences were statistically significant. These results suggest that TubA intervention effectively alleviated chronic stress-induced postoperative restenosis.

### 3.4 HDAC6 inhibition restores autophagy, suppresses apoptosis and mitigates vascular inflammation

To detect the effect of HDAC6 inhibition on the proliferative pathway, the injured carotid arteries were further subjected to WB assay, and the results are shown in Figure 6C. Compared with the solvent intraperitoneal injection control group, administration of TubA significantly inhibited the phosphorylation level of HDAC6 in the injured carotid arteries under the chronic stress condition(*p* < 0.05) (Figure 6D), and the difference was statistically significant.

We also examined the expression of autophagy and apoptosis-related pathway proteins, and the results are shown in Figures 7A,F. Compared with the solvent intraperitoneal injection control group, TubA administration decreased the phosphorylation levels of PI3 K(*p* < 0.01) (Figure 7B), AKT(*p* < 0.05) (Figure 7C), and mTOR(*p* < 0.01) (Figure 7D), elevated autophagy marker LC3B/A level(*p* < 0.05) (Figure 7E), decreased the protein level of pro-apoptotic factor Bax(*p* < 0.01) (Figure 7G) and increased the protein expression level of anti-apoptotic protein Bcl-2(*p* < 0.05) (Figure 7H), and the differences were all statistically significant. The above suggests that TubA activates autophagy and inhibits apoptosis by inhibiting the PI3 K/Akt/mTOR pathway.

**Figure 7:**
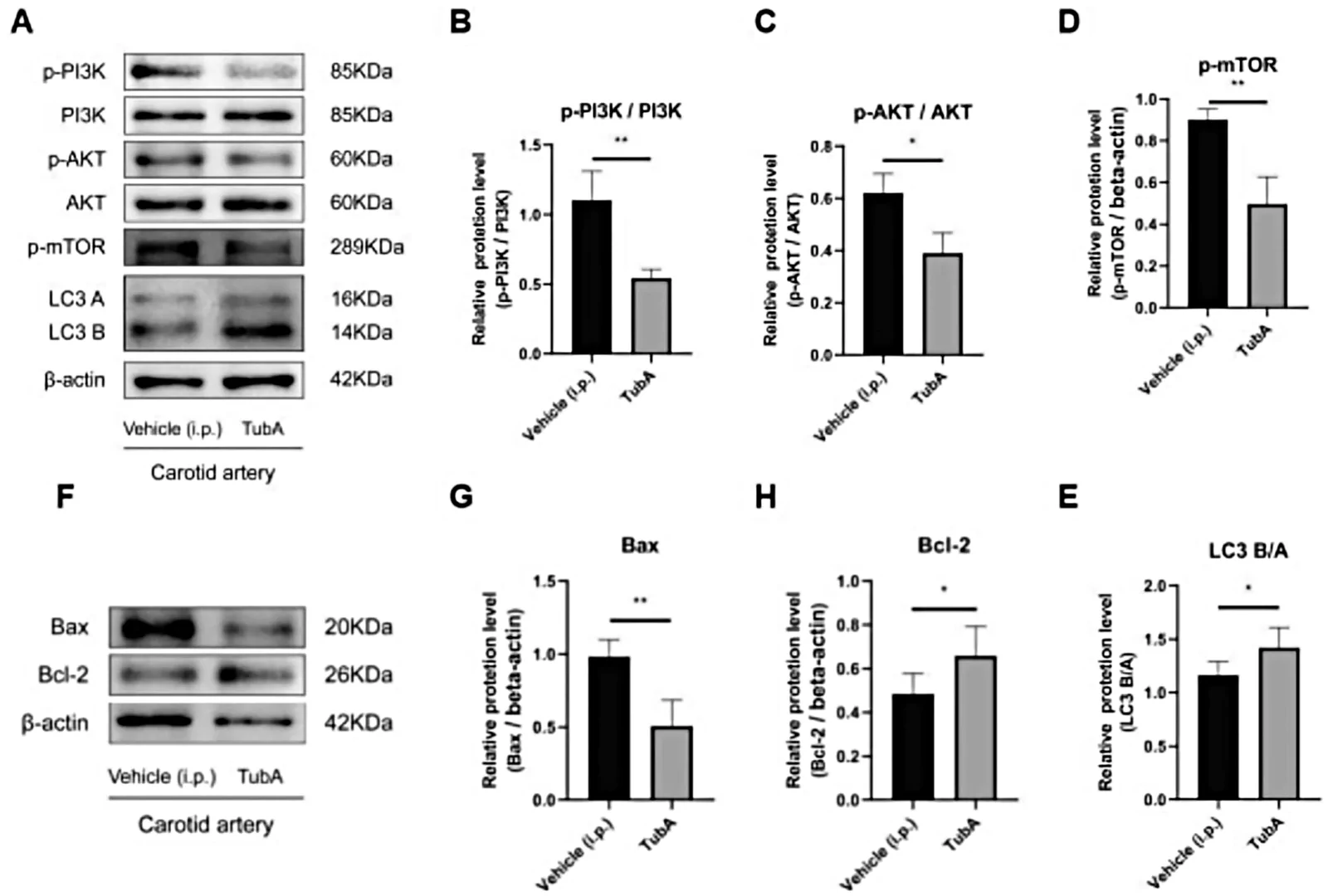
Autophagy and apoptosis-related protein expression in TubA-treated carotid tissues (post-operative Day 4, *n* = 5). **(A–E)** WB bands and quantitative statistics of PI3 K, *p*-PI3 K, AKT, *p*-AKT, mTOR, *p*-mTOR and LC3B/A; **(F–H)** Immunoblots and gray value quantification of pro-apoptotic Bax and anti-apoptotic Bcl-2. *p < 0.05, **p < 0.01.

Collectively, these findings demonstrate that HDAC6 inhibition mimics the protective effects of formononetin against chronic stress-induced postoperative restenosis. Specifically, TubA(tubastatin A) alleviates chronic stress-mediated impairment of carotid artery autophagy and reduces apoptosis, thereby attenuating stress-aggravated neointimal hyperplasia following vascular intervention.

To verify whether inhibition of HDAC6 could play a role in reducing inflammation, WB assay was performed on the injured carotid arteries, and the results are shown in Figure 6E. Compared with the solvent intraperitoneal injection control group, TubA administration significantly suppressed the protein expression level of NLRP3 in the injured carotid arteries under the conditions of chronic stress(*p* < 0.01) (Figure 6F), and reduced the protein expression of IL-6(*p* < 0.05) (Figure 6G), both differences were statistically significant.

### 3.5 FMN modulates autophagy, apoptosis and NLRP3/IL-6 inflammation through HDAC6-dependent signaling

A previous study by our group also suggested that chronic stress can lead to impaired autophagy in depressed mice (23), therefore, we examined autophagy-related pathways in injured carotid artery tissues of mice exposed to chronic stress: PI3 K, AKT, mTOR, and autophagy markers LC3B/A, and apoptosis-related proteins: Bax, Bcl-2) expression.

WB results showed (Figures 8A,F) that compared with the solvent gavage control group, FMN decreased the phosphorylation levels of PI3 K(*p* < 0.01) (Figure 8B), AKT(*p* < 0.01) (Figure 8C), and mTOR(*p* < 0.05) (Figure 8D), increased the level of autophagy markers LC3B/A(*p* < 0.01) (Figure 8E), decreased the level of pro-apoptotic factor Bax(*p* < 0.05) (Figure 8G) and increased the expression level of anti-apoptotic protein Bcl-2 expression level(*p* < 0.01) (Figure 8H), and the differences were statistically significant. It suggests that FMN activates autophagy and inhibits apoptosis by inhibiting PI3 K phosphorylation and inactivating it, which in turn blocks PI3 K/Akt/mTOR signaling.

**Figure 8:**
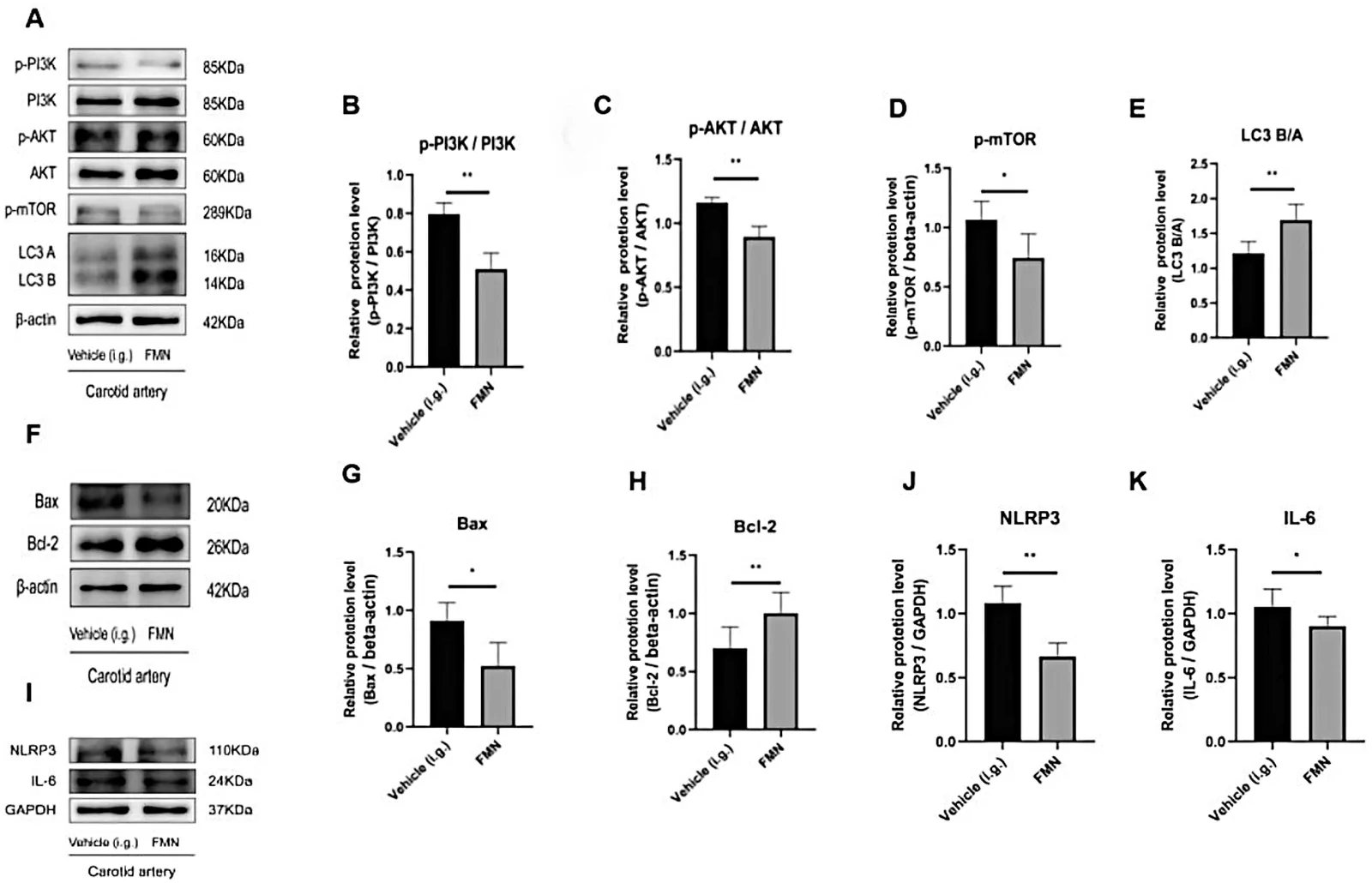
Autophagy, apoptosis and inflammatory protein expression in FMN-treated carotid tissues (post-operative Day 4, *n* = 5). **(A–E)** Immunoblot profiles and quantification of PI3 K/Akt/mTOR autophagic signaling and LC3B/A; **(F–H)** Band images and statistical analysis of Bax and Bcl-2; **(I–K)** WB detection and quantification of NLRP3 inflammasome and IL-6 pro-inflammatory cytokine.*p < 0.05, **p < 0.01.

What has been demonstrated is that chronic stress is associated with increased systemic inflammation (28). Inflammation and oxidative stress are considered to be the main risk factors that increase the occurrence of stent-related complications such as restenosis after percutaneous coronary intervention(PCI) (29). To test whether FMN can attenuate the expression of inflammation-related factors in chronically stress-induced injured arteries, we examined the protein expression of the inflammatory vesicle NLRP3 and the inflammatory factor interleukin-6(IL-6) using WB. As shown in Figures 7I–K, compared with the solvent gavage control group, FMN administration significantly reduced the protein expression of the inflammatory vesicle NLRP3(*p* < 0.01) as well as the pro-inflammatory factor IL-6(*p* < 0.05), both of which were statistically significant.

### 3.6 FMN alleviates vascular senescence and inhibits VSMC proliferation, migration and apoptosis *in vitro*

#### 3.6.1 FMN attenuates chronic stress-induced aortic senescence

Chronic stress has been reported to accelerate diet-induced vascular senescence and atherosclerotic plaque destabilization (30–32). To further observe whether FMN had an ameliorative effect on chronic stress-induced vascular senescence, we performed *β*-galactosidase(*β*-Gal) staining of the aorta of mice in each group of mice subjected to chronic stress for 14 days postoperatively to assess vascular senescence. As shown in Figure 9A, under chronic stress, the aortas of mice in the solvent gavage control group and solvent intraperitoneal injection control group exhibited a severe degree of senescence, whereas the degree of aortic senescence in mice in both the FMN gavage group and the TubA intraperitoneal injection group was significantly attenuated in comparison with their respective control groups.

**Figure 9:**
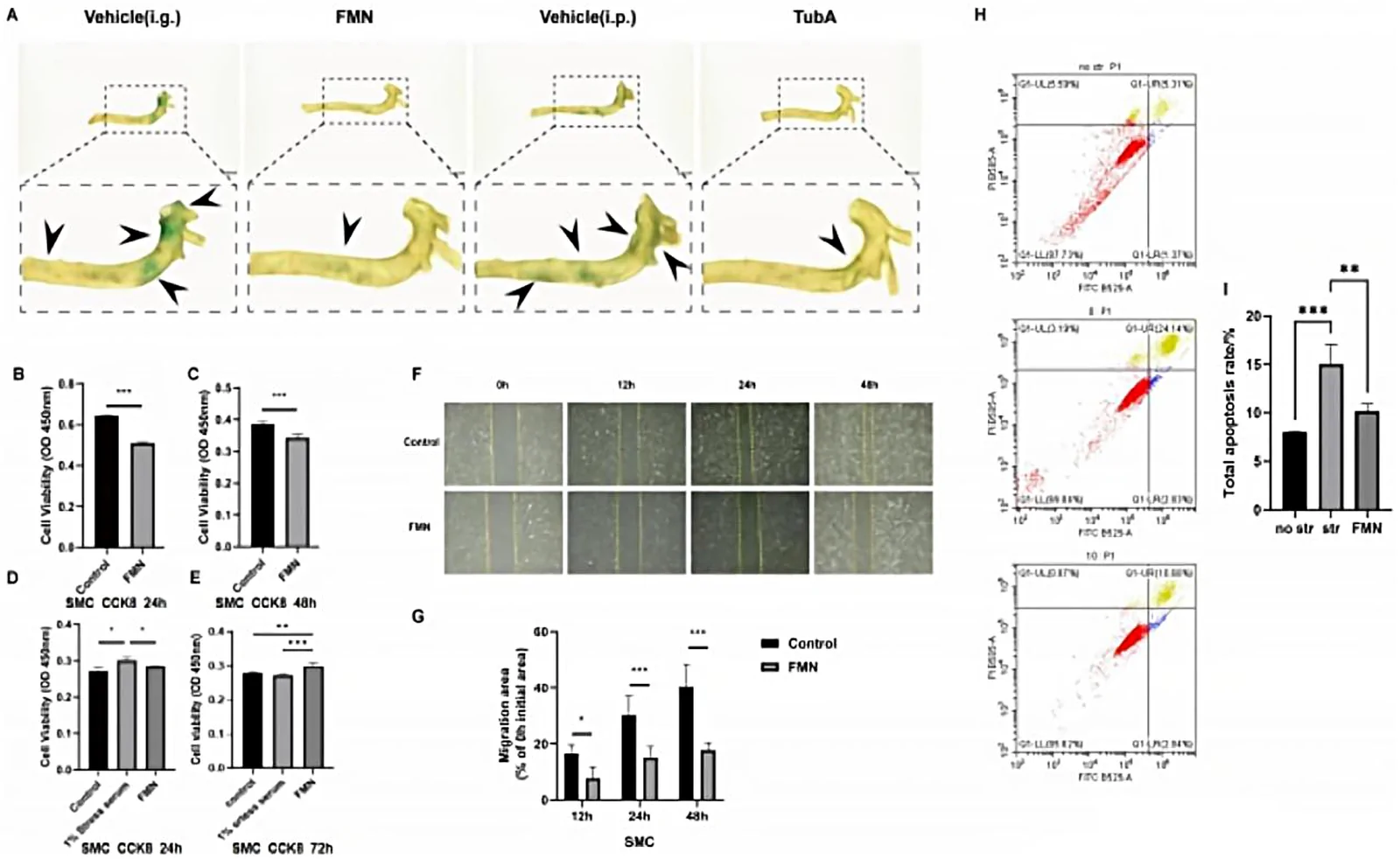
Aortic senescence staining and *in vitro* VSMC functional assays. **(A)** Representative *β*-Gal staining of mouse aortas, with arrows marking blue senescence-positive regions; **(B,C)** CCK-8 OD values reflecting basal VSMC proliferation at 24 h and 48 h; **(D,E)** CCK-8 proliferation data of VSMCs cultured with CUMS mouse serum with or without FMN treatment (24 h, 72 h); **(F,G)** Bright-field scratch wound images and quantitative migration rate statistics at 0, 12, 24 and 48 h; **(H,I)** Flow cytometric Annexin V/PI apoptosis scatter plots and total apoptosis rate quantification. Control: drug-solvent control group, no str: group of no stressed mice serum added to the medium by 1%, 1% stress serum(str): group of stressed mice serum added to the medium by 1%, FMN, formononetin treatment group(10μM) with group of stressed mice serum added to the medium by 1%. *p < 0.05, **p < 0.01, ***p < 0.001.

#### 3.6.2 FMN suppresses VSMC proliferation and migration

Abnormal activation, proliferation, and migration of VSMC are important links in the occurrence of postoperative restenosis, we demonstrated *in vivo* experiments that FMN can effectively alleviate postoperative restenosis by inhibiting the HDAC6 pathway, to further explore the effect of FMN on VSMC proliferation and migration, we conducted *in vitro* experiments. The cell viability of SMC treated with FMN and drug solvent under 1% FBS for 24 h and 48 h, respectively, was detected using CCK-8. The results showed (Figures 9B,C) that the cell proliferation viability of the FMN(10*μ*M)-treated group was significantly reduced compared with that of the control group at both 24 h and 48 h(*p* < 0.001 and *p* < 0.01, respectively), indicating that FMN was able to inhibit the effect of VSMC proliferation (33).

To investigate the molecular mechanism of chronic stress-mediated postoperative restenosis, in this study, SMC was cultured for 24 h and 72 h in medium with and without the addition of 1% stress 4-week mouse serum at the same time (34), and the results showed (Figures 9D,E) that at 24 h, the cell proliferative activity was significantly elevated in the group with 1% added stress mouse serum compared to the control group(*p* < 0.05), while the FMN(10μM) treatment significantly alleviated this abnormal increase in cell proliferative activity(*p* < 0.05); at 48 h, the FMN(10μM) treatment significantly alleviated this abnormal increase in cell proliferative activity(*p* < 0.001). It is suggested that FMN can inhibit the proliferation of SMC *in vitro* and inhibit the proliferation of vascular smooth muscle cells induced by serum in chronically stressed mice.

#### 3.6.3 Formononetin inhibits vascular smooth muscle cell(VSMC) migration

To observe the effect of FMN on SMC migration, this study conducted cell scratching experiments, microscopic observation and image uptake were performed at 0 h, 12 h, 24 h, and 48 h after scratching, and the cell migration area under each time point was analyzed using Image J software to calculate the cell migration rate. The results showed (Figure 9F) that the migration rate of cells in the FMN(10 μM)-treated group was significantly reduced compared with that of the control group at 12 h, 24 h, and 48 h of incubation, and the differences were all statistically significant(*p* < 0.05, *p* < 0.001, *p* < 0.001), indicating that FMN could inhibit SMC migration *in vitro*.

#### 3.6.4 Formononetin inhibits vascular smooth muscle cell(VSMC) apoptosis

To observe the effect of FMN on smooth muscle cell (SMC) apoptosis, this study conducted an Annexin V/PI assay on the no str, str, and FMN groups. The results showed (Figure 9H) that compared with the no str group, the total apoptosis rate in the str group was significantly increased, while the total apoptosis rate in the FMN (10 μM) treatment group was significantly decreased. The differences were statistically significant (*p* < 0.01, *p* < 0.001) (Figure 9I), indicating that FMN can inhibit SMC apoptosis *in vitro*.

## 4 Discussion

Postoperative restenosis is a complex process frequently occurring after angioplasty, bypass grafting, and endarterectomy of the coronary and peripheral arteries. It remains a major limitation to the long-term efficacy of endovascular interventions and contributes significantly to cardiovascular mortality (35). The underlying mechanisms primarily involve post-procedural endothelial denudation, intimal injury, persistent vascular inflammation, and oxidative stress, which collectively drive aberrant activation, proliferation, and migration of vascular smooth muscle cells, ultimately leading to neointimal hyperplasia and/or neoatherosclerosis. Notably, chronic stress has been shown to exacerbate endothelial damage, chronic inflammation, lipid metabolism disorders, and atherosclerosis (3), chronic stress background induces myocardial pathological hypertrophy in mice, and there are extensive gender differences in pathological remodeling of angiotensin II(AngII), and many of the up-regulated genes are regulated by estradiol (36). FMN, as an isoflavonoid, belongs to a kind of phytoestrogen, and several *in vivo* and *ex vivo* studies have confirmed that FMN produces anticardiovascular disease and anti-hypertensive effects (37); neuroprotective effects to improve Alzheimer’s disease and antidepressant (38); and inhibitory properties on tumor cell proliferation (39), among others. However, the role of FMN in preventing postoperative restenosis under chronic stress conditions has not yet been reported. To address this gap, we established a dual mouse model of chronic stress and carotid artery injury to investigate whether FMN attenuates restenosis and to explore the underlying mechanisms.

To assess the impact of FMN on restenosis under chronic stress, carotid artery sections from mice on postoperative day 14 were stained with H&E, and the neointimal area as well as the neointima-to-media ratio were measured. In line with earlier findings from our group (40), chronic stress markedly increased both the neointimal area and the neointima-to-media ratio, reflecting exacerbated restenosis of the injured right carotid artery. In contrast, FMN treatment significantly reduced these parameters, indicating that FMN attenuates chronic stress-induced restenosis. In addition to its known activities, FMN has been reported to confer beneficial effects in multiple vascular insufficiency-associated pathological conditions. For instance, in apoE-deficient mice fed a high-fat diet, FMN administration significantly reduced the area of atherosclerotic lesions in the aortic arch and aortic root sinus, and improved plaque stability through compositional modulation (22). In a disease model of inferior vena cava stenosis-deep venous thrombosis(DVT) in rats, FMN reduced DVT-induced vascular endothelial injury by attenuating DVT (41). The present study suggests that FMN also has potential in the treatment of cardiovascular disease with restenosis after vascular injury in the context of chronic stress.

In response to vascular injury, migration and proliferation of vascular smooth muscle cells from the mid-membrane to the intima are key events in the development of restenotic lesions (42). An important factor in vascular smooth muscle cell migration is the degradation of the basement membrane and surrounding extracellular matrix (43). Previous studies have shown that histone deacetylase 6 is associated with many key signaling pathways, such as HDAC6 and the PI3 K/AKT/mTOR signaling pathway (13), and that histone deacetylase 6 is a unique histone deacetylase with two functional catalytic structural domains(DD1 and DD2) and a ZnF-UBP structural domain(ubiquitin-binding structural domain), which regulates many biological processes, including gene expression, cell motility, immune responses, and degradation of misfolded proteins, HDAC6 is also required to regulate the proliferation and migration of various types of cancer cells (35). Inhibition of HDAC6 activation to stimulate VSMC migration and proliferation in vascular remodeling and neointimal hyperplasia (34).In the present study, we found that FMN treatment significantly reduced HDAC6 protein levels in injured carotid arteries under chronic stress, concomitant with marked attenuation of neointimal hyperplasia. This effect was phenocopied by administration of the selective HDAC6 inhibitor TubA.

The release of vasoactive and pro-inflammatory mediators during chronic inflammation promotes phenotypic switching of smooth muscle cells, which in turn contributes to intimal hyperplasia and vascular lumen narrowing. In parallel, chronic stress has been demonstrated to elicit a systemic inflammatory response in murine models (30). *In vivo* administration of FMN markedly attenuated the protein levels of the NLRP3 inflammasome as well as the pro-inflammatory cytokines IL-6 and p38 in injured carotid arteries under chronic stress. Administration of the HDAC6-specific inhibitor TubA similarly reduced NLRP3 and IL-6 expression, phenocopying the effect of FMN. Of note, formononetin has previously been reported to ameliorate house dust mite-induced airway inflammation in asthmatic mice by facilitating epithelial barrier restoration, with inhibition of the ESR1/NLRP3/Caspase-1 signaling proposed as a potential underlying mechanism (44). Formononetin significantly ameliorated the inflammatory response and apoptosis in LPS-induced inflammatory injury in neuronal cells through the NF-*κ*B/NLRP3 signaling pathway (45). Recently, it was reported that HDAC6 enhanced the interaction between the lysosomal regulator complex and NLRP3, leading to the activation of NLRP3 inflammasome, which ameliorated cardiac injury in myocardial tissues of rats with dilated cardiomyopathy. And in injured cardiomyocytes, HDAC6 silencing also reduced NLRP3 inflammasome activation and apoptosis, but enhanced cellular autophagy (46).

Autophagy is an evolutionarily conserved catabolic process that is primarily responsible for the recycling of unnecessary cellular organs and excess nutrients, and the elimination of metabolic wastes and intracellular pathogens. There is growing evidence that chronic stress leads to autophagy dysfunction (16). Chronic stress has been shown not only to accelerate atherosclerosis in apoE⁻/⁻ mice but also to impair arterial autophagic flux, as evidenced by decreased LC3 and increased p62 protein levels. In the present study, we observed that FMN ameliorated chronic stress-induced autophagy defects by inhibiting PI3 K/AKT/mTOR signaling activation, as reflected by increased LC3B/LC3A ratio (a marker of autophagy) and restored autophagic homeostasis. Concurrently, FMN treatment reduced the expression of the pro-apoptotic factor Bax and increased that of the anti-apoptotic protein Bcl-2. Mechanistically, our data indicate that FMN modulates Akt signaling in a partial and cell context-dependent manner under chronic stress conditions, thereby re-establishing autophagic flux. Notably, the observed anti-apoptotic phenotype-characterized by an elevated Bcl-2/Bax ratio-likely arises as a secondary consequence of restored autophagic function, rather than from direct suppression of Akt activity. The regulatory role of FMN in autophagy and apoptosis has been reported, and formononetin was able to attenuate ischemia/reperfusion-induced apoptosis in senescent cells by promoting autophagy (47).FMN promoted lysosome biogenesis and fusion of autophagosomes with lysosomes, which attenuated autophagic flux blockage, further induced lipophagy, and ameliorated hepatic steatosis (48). In addition, it has been shown that HDAC6’s are also involved in the regulation of a variety of processes including cell proliferation, cell differentiation, and apoptosis (49). HDAC6 was upregulated in rats with dilated cardiomyopathy, and HDAC6 silencing enhanced cardiomyocyte autophagy and suppressed NLRP3 inflammasome activation, which ameliorated the cardiac injury in cardiac myocyte tissues of dilated cardiomyopathic rats. In injured cardiomyocytes, HDAC6 silencing also reduced NLRP3 inflammasome activation and apoptosis but enhanced cellular autophagy (50).

The inflammatory microenvironment brought about by chronic stress and vascular injury affects many cellular physiological processes such as cellular senescence. Cellular senescence fails to function properly and secretes inflammatory cytokines and degradation proteins, inducing a further vicious cycle of senescence and inflammation (51). It has been reported that chronic variable stress accelerates diet-induced vascular senescence and atherosclerotic plaque growth and instability (30–32). Autophagy is also closely related to the onset of aging, and disruption of autophagy accelerates cellular aging and death and promotes the onset of aging. The results of the present study found that FMN alleviated chronic stress-accelerated senescence in mouse aorta, and administration of TubA produced similar effects, which may be attributed to its anti-inflammatory, anti-apoptotic, and autophagy activation effects.

This study integrated network pharmacology with animal experiments to preliminarily elucidate the mechanism by which FMN ameliorates chronic stress-induced vascular intimal hyperplasia and comorbid anxiety and depression. Network pharmacology analysis identified the overlapping targets between FMN and cardiovascular disease–depression comorbidity (21). Molecular docking further confirmed that FMN could stably bind to key proteins such as HDAC6, Bcl-2, IL-6 and AKT1, suggesting that FMN possesses a multi-target pharmacological basis for synergistically regulating psychosomatic injury. *In vivo* experiments demonstrated that chronic stress markedly exacerbated abnormal intimal hyperplasia following vascular injury and induced vascular aging, accompanied by anxiety- and depression-like behaviors in mice. FMN intervention significantly alleviated the emotional disturbances, suppressed vascular intimal thickening, downregulated HDAC6 expression, restored autophagic flux, and inhibited excessive apoptosis in these model mice. Moreover, the HDAC6 inhibitor TubA recapitulated the vascular protective effects of FMN. These experimental findings are highly consistent with the inflammation- and apoptosis-regulatory pathways enriched by network pharmacology, further supporting that FMN attenuates chronic stress-mediated vascular remodeling and psychosomatic comorbidity by targeting HDAC6, rebalancing autophagy and apoptosis, and suppressing inflammatory responses (52). In conclusion, FMN confers dual benefits of vascular protection and mood improvement, providing new theoretical and experimental evidence from a natural product for the comprehensive prevention and treatment of chronic stress-related cardiovascular diseases comorbid with emotional disorders.

The limitations of the modern biomedical model do not explain and solve all medical problems. In the 1970s, the American scholar Engel proposed a new medical model, i.e., the biopsychosocial medical model, which points out that human beings are the unity of biology, society, and psychology, and emphasizes the treatment of mind and body together. To focus on the wholeness and harmony of mind-body health, the present study investigated the psychological and physiological changes of chronic stress on mice in the postoperative restenosis model. Depression is one of the most common serious central nervous system disorders, negatively affecting people’s social life, workability, and health. According to the World Health Organization, this mental disorder is expected to become a major disease by 2030 (53). The previous study of our group showed that chronic unpredictable mild stress can induce depression-like symptoms in mice (23), to observe the effects of chronic stress in different model mice, this study conducted a sucrose preference rate test to assess depression-like symptoms of pleasure deficit, immobility time in forced swimming to assess depression-like symptoms of behavioral despair, percentage of entries in the central area of the absenteeism experiment, and percentage of stay time in the central area to assess depression-like symptoms of anxiety. Percentage of time spent in the central zone to assess anxiety-like symptoms. The results showed that chronic stress also induced anxiety and depression-like symptoms in mice with the carotid intima-media injury model, and FMN administration was effective in improving anxiety and depression-like symptoms in both models. FMN has been reported to significantly increase sucrose preference and shorten immobilization time in a forced swimming experiment in a mouse model of depression induced by chronic corticosterone(CORT) injection, and to upregulate glucocorticoid receptor(GR) and brain-derived neurotrophic factor(BDNF). Brain-derived neurotrophic factor(BDNF) protein expression levels, prevented CORT-induced neuronal damage and promoted hippocampal neurogenesis (54). Network pharmacological studies have shown that FMN targeting IL-6, MAPK 1, signal transducer and activator of transcription 3(STAT 3), and Transcription Factor AP-1/JUN Targeted regulation can play an antidepressant role (55). FMN can inhibit the release of inflammatory factors by blocking the NF-*κ*B pathway and improve the cognitive and behavioral abilities of aged rats in the chronic unpredictable mild stress(CUMS) model (56). This may help to explain the molecular mechanisms underlying the ameliorative effects of FMN on anxiety and depression-like symptoms in chronic stress-induced arterial injury mice in the present study. The natural flavonoid rutin inhibited cellular HDAC6 activity without inducing any toxicity and significantly increased the level of *α*-microtubulin acetylation in motor neuron-like cells, which is considered a promising therapeutic target for neurodegenerative disorders, suggesting that targeted therapy against HDAC6 may also have the potential to ameliorate neuropsychiatric disorders.

Preliminary gradient experiments using 5, 10, and 20 mg/kg FMN demonstrated that the 10 mg/kg dosage produced the most potent regulatory effect on autophagy, which was therefore selected as the working intervention dose for subsequent formal experiments. One notable limitation of the present study is that female mice were only included as untreated blank controls, without corresponding FMN-treated female cohorts. Given that the primary objective of this work was to preliminarily characterize FMN’s protective actions against chronic stress-induced intimal hyperplasia, we did not systematically assess potential sex-specific pharmacological responses. As a phytoestrogen capable of binding estrogen receptors, FMN may elicit divergent biological effects across sexes. Future investigations incorporating both male and female FMN-treated animals are warranted to dissect sex-dependent disparities and the underlying estrogen receptor-mediated mechanisms.

## 5 Conclusion

Integrated findings from this study demonstrate that chronic stress exacerbates intimal hyperplasia in injured arteries, leading to postoperative restenosis, while concurrently inducing anxiety- and depression-like behaviors. Formononetin ameliorated both neurobehavioral disturbances and vascular pathology in chronic stress-exposed mice with arterial injury. The therapeutic effects are potentially mediated through HDAC6 suppression, which consequently inhibits vascular smooth muscle cell proliferation/migration, restores autophagic flux in injured carotid tissues, and attenuates inflammatory responses and apoptosis, thereby mitigating stress-aggravated neointimal hyperplasia and vascular restenosis. Notably, FMN also alleviated chronic stress-accelerated aortic senescence. Given the dual detrimental effects of chronic stress on both physical and mental health, formononetin exhibits promising “dual-organ” therapeutic potential for ameliorating cerebrovascular and psychiatric comorbidities in stress-associated carotid injury models.

## Data Availability

The datasets presented in this study can be found in online repositories. The names of the repository/repositories and accession number(s) can be found in the article/Supplementary Material.

## References

[1] Osborne MT Shin LM Mehta NN Pitman RK Fayad ZA Tawakol A. Disentangling the links between psychosocial stress and cardiovascular disease. Circ Cardiovasc Imaging. (2020) 13:e010931. 10.1161/CIRCIMAGING.120.010931 32791843 PMC7430065

[2] Razzoli M Bartolomucci A. The dichotomous effect of chronic stress on obesity. Trends Endocrinol Metab. (2016) 27:504–15. 10.1016/j.tem.2016.04.007 27162125 PMC4912918

[3] Gao S Wang X Meng LB Zhang YM Luo Y Gong T. Recent progress of chronic stress in the development of atherosclerosis. Oxid Med Cell Longev. (2022) 2022:4121173. 10.1155/2022/4121173 35300174 PMC8923806

[4] Milligan AA Porter T Quek H White A Haynes J Jackaman C. Chronic stress and Alzheimer’s disease: the interplay between the hypothalamic-pituitary-adrenal axis, genetics and microglia. Biol Rev Camb Philos Soc. (2021) 96:2209–28. 10.1111/brv.12750 34159699

[5] Torpy JM Lynm C Glass RM. JAMA Patient page chronic stress and the heart. JAMA. (2007) 298:1722. 10.1001/jama.298.14.1722 17925527

[6] Hong H Ji M Lai D. Chronic stress effects on tumor: pathway and mechanism. Front Oncol. (2021) 11:738252. 10.3389/fonc.2021.738252 34988010 PMC8720973

[7] Kelly RR McDonald LT Jensen NR Sidles SJ LaRue AC. Impacts of psychological stress on osteoporosis: clinical implications and treatment interactions. Front Psychiatry. (2019) 10:200. 10.3389/fpsyt.2019.00200 31024360 PMC6465575

[8] Di Cesare M Perel P Taylor S Kabudula C Bixby H Gaziano TA. The heart of the world. Glob Heart. (2024) 19:11. 10.5334/gh.1288 38273998 PMC10809869

[9] Roth GA Mensah GA Johnson CO Addolorato G Ammirati E Baddour LM. Global burden of cardiovascular diseases and risk factors, 1990–2019: update from the GBD 2019 study. J Am Coll Cardiol. (2020) 76:2982–3021. 10.1016/j.jacc.2020.11.010 33309175 PMC7755038

[10] Khan Y Verhaeghe N Devleesschauwer B Cavillot L Gadeyne S Pauwels NS. Impact of the COVID-19 pandemic on delayed care of cardiovascular diseases in Europe: a systematic review. Lancet. (2023) 402(Suppl 1):S61. 10.1016/S0140-6736(23)02117-7 37997105

[11] Huynh D Heo KS. Role of mitochondrial dynamics and mitophagy of vascular smooth muscle cell proliferation and migration in progression of atherosclerosis. Arch Pharm Res. (2021) 44:1051–61. 10.1007/s12272-021-01360-4 34743301

[12] Texakalidis P Tzoumas A Giannopoulos S Jonnalagadda AK Jabbour P Rangel-Castilla L. Risk factors for restenosis after carotid revascularization: a meta-analysis of hazard ratios. World Neurosurg. (2019) 125:414–24. 10.1016/j.wneu.2019.02.065 30822589

[13] Wu H Cheng XW Hu L Takeshita K Hu C Du Q. Cathepsin S activity controls injury-related vascular repair in mice via the TLR2-mediated p38MAPK and PI3K-akt/p-HDAC6 signaling pathway. Arterioscler Thromb Vasc Biol. (2016) 36:1549–57. 10.1161/ATVBAHA.115.307110 27365406 PMC4961274

[14] Çakır I Hadley CK Pan PL Bagchi RA Ghamari-Langroudi M Porter DT. Histone deacetylase 6 inhibition restores leptin sensitivity and reduces obesity. Nat Metab. (2022) 4:44–59. 10.1038/s42255-021-00515-3 PMC8892841 35039672

[15] Magupalli VG Negro R Tian Y Hauenstein AV Di Caprio G Skillern W. HDAC6 Mediates an aggresome-like mechanism for NLRP3 and pyrin inflammasome activation. Science. (2020) 369:eaas8995. 10.1126/science.aas8995 32943500 PMC7814939

[16] Li N Zhang RX Xie XJ Gu HF. Autophagy in chronic stress induced atherosclerosis. Clin Chim Acta. (2020) 503:70–5. 10.1016/j.cca.2020.01.006 31945340

[17] Oda H Annibaldi A Kastner DL Aksentijevich I. Genetic regulation of cell death: insights from autoinflammatory diseases. Annu Rev Immunol. (2025) 43:313–42. 10.1146/annurev-immunol-090222-105848 40279314

[18] Zhou Z Li T Qin H Wang X He S Fan Z. Acacetin as a natural cardiovascular therapeutic: mechanisms and preclinical evidence. Front Pharmacol. (2025) 16:1493981. 10.3389/fphar.2025.1493981 40255574 PMC12006078

[19] Chen JJ Shang XY Han FY Zhang Y Zhao D Yao GD. Network pharmacology predicted HDAC6 as a potential target of flavones from Daphne giraldii on hepatocellular carcinoma. Nat Prod Res. (2021) 35:3171–5. 10.1080/14786419.2019.1693563 31741408

[20] Hao Y Miao J Liu W Peng L Chen Y Zhong Q. Formononetin protects against cisplatin-induced acute kidney injury through activation of the PPAR*α*/Nrf2/HO-1/NQO1 pathway. Int J Mol Med. (2021) 47:511–22. 10.3892/ijmm.2020.4805 33416097 PMC7797437

[21] Xiao H Qin X Wan J Li R. Pharmacological targets and the biological mechanisms of formononetin for Alzheimer’s disease: a network analysis. Med Sci Monit. (2019) 25:4273–7. 10.12659/MSM.916662 31175839 PMC6580864

[22] Ma C Xia R Yang S Liu L Zhang J Feng K. Formononetin attenuates atherosclerosis via regulating interaction between KLF4 and SRA in apoE(-/-) mice. Theranostics. (2020) 10:1090–106. 10.7150/thno.38115 31938053 PMC6956811

[23] Zhang Y Wu H Xu C Li S Hu Y Zhang Z. (-)-Epigallocatechin gallate alleviates chronic unpredictable mild stress-induced depressive symptoms in mice by regulating the mTOR autophagy pathway and inhibiting NLRP3 inflammasome activation. Food Sci Nutr. (2024) 12:459–70. 10.1002/fsn3.3761 38268911 PMC10804105

[24] He LW Zeng L Tian N Li Y He T Tan DM. Optimization of food deprivation and sucrose preference test in SD rat model undergoing chronic unpredictable mild stress. Animal Model Exp Med. (2020) 3:69–78. 10.1002/ame2.12107 32318662 PMC7167236

[25] Tang J Liang X Dou X Qi Y Yang C Luo Y. Exercise rather than fluoxetine promotes oligodendrocyte differentiation and myelination in the hippocampus in a male mouse model of depression. Transl Psychiatry. (2021) 11:622. 10.1038/s41398-021-01747-3 34880203 PMC8654899

[26] Tunc-Ozcan E Peng CY Zhu Y Dunlop SR Contractor A Kessler JA. Activating newborn neurons suppresses depression and anxiety-like behaviors. Nat Commun. (2019) 10:3768. 10.1038/s41467-019-11641-8 31434877 PMC6704083

[27] Wang H Meng X Piao L Inoue A Xu W Yu C. Cathepsin S deficiency mitigated chronic stress-related neointimal hyperplasia in mice. J Am Heart Assoc. (2019) 8:e011994. 10.1161/JAHA.119.011994 31296090 PMC6662117

[28] Dhabhar FS. Effects of stress on immune function: the good, the bad, and the beautiful. Immunol Res. (2014) 58:193–210. 10.1007/s12026-014-8517-0 24798553

[29] Clare J Ganly J Bursill CA Sumer H Kingshott P De Haan JB. The mechanisms of restenosis and relevance to next generation stent design. Biomolecules. (2022) 12:430. 10.3390/biom12030430 35327622 PMC8945897

[30] Lei Y Yang G Hu L Piao L Inoue A Jiang H. Increased dipeptidyl peptidase-4 accelerates diet-related vascular aging and atherosclerosis in ApoE-deficient mice under chronic stress. Int J Cardiol. (2017) 243:413–20. 10.1016/j.ijcard.2017.05.062 28549747

[31] Piao L Zhao G Zhu E Inoue A Shibata R Lei Y. Chronic psychological stress accelerates vascular senescence and impairs ischemia-induced neovascularization: the role of dipeptidyl peptidase-4/glucagon-like peptide-1-adiponectin axis. J Am Heart Assoc. (2017) 6:e006421. 10.1161/JAHA.117.006421 28963101 PMC5721852

[32] Yang G Lei Y Inoue A Piao L Hu L Jiang H. Exenatide mitigated diet-induced vascular aging and atherosclerotic plaque growth in ApoE-deficient mice under chronic stress. Atherosclerosis. (2017) 264:1–10. 10.1016/j.atherosclerosis.2017.07.014 28734203

[33] Wang Q Ou Y Hu G Wen C Yue S Chen C. Naringenin attenuates non-alcoholic fatty liver disease by down-regulating the NLRP3/NF-*κ*B pathway in mice. Br J Pharmacol. (2020) 177:1806–21. 10.1111/bph.14938 31758699 PMC7070172

[34] Yue X Piao L Wang H Huang Z Meng X Sasaki T. Cathepsin K deficiency prevented kidney damage and dysfunction in response to 5/6 nephrectomy injury in mice with or without chronic stress. Hypertension. (2022) 79:1713–23. 10.1161/HYPERTENSIONAHA.122.19137 35726642 PMC9278705

[35] Lee MS Banka G. In-stent restenosis. Interv Cardiol Clin. (2016) 5:211–20. 10.1016/j.iccl.2015.12.006 28582205

[36] McLellan MA Skelly DA Dona M Squiers GT Farrugia GE Gaynor TL. High-Resolution transcriptomic profiling of the heart during chronic stress reveals cellular drivers of cardiac fibrosis and hypertrophy. Circulation. (2020) 142:1448–63. 10.1161/CIRCULATIONAHA.119.045115 32795101 PMC7547893

[37] Bankar GR Nayak PG Bansal P Paul P Pai KS Singla RK. Vasorelaxant and antihypertensive effect of Cocos nucifera linn. Endocarp on isolated rat thoracic aorta and DOCA salt-induced hypertensive rats. J Ethnopharmacol. (2011) 134:50–4. 10.1016/j.jep.2010.11.047 21129472

[38] Tian J Wang XQ Tian Z. Focusing on formononetin: recent perspectives for its neuroprotective potentials. Front Pharmacol. (2022) 13:905898. 10.3389/fphar.2022.905898 35712702 PMC9196267

[39] Kim C Lee SG Yang WM Arfuso F Um JY Kumar AP. Formononetin-induced oxidative stress abrogates the activation of STAT3/5 signaling axis and suppresses the tumor growth in multiple myeloma preclinical model. Cancer Lett. (2018) 431:123–41. 10.1016/j.canlet.2018.05.038 29857127

[40] Hu Y. *Association of cathepsin K with sex-related neointimal hyperplasia in chronic stress-induced mice* (Master's thesis). Guilin Medical University, Guilin, China (2021).

[41] Zhou Z Zhou H Zou X Wang X Yan M. Formononetin regulates endothelial nitric oxide synthase to protect vascular endothelium in deep vein thrombosis rats. Int J Immunopathol Pharmacol. (2022) 36:3946320221111117. 10.1177/03946320221111117 35731855 PMC9228649

[42] Lin SJ Yen HT Chen YH Ku HH Lin FY Chen YL. Expression of interleukin-1 beta and interleukin-1 receptor antagonist in oxLDL-treated human aortic smooth muscle cells and in the neointima of cholesterol-fed endothelia-denuded rabbits. J Cell Biochem. (2003) 88:836–47. 10.1002/jcb.10431 12577317

[43] Burns-Kurtis CL Olzinski AR Needle S Fox JH Capper EA Kelly FM. Cathepsin S expression is up-regulated following balloon angioplasty in the hypercholesterolemic rabbit. Cardiovasc Res. (2004) 62:610–20. 10.1016/j.cardiores.2004.02.002 15158154

[44] Zhang L Wu Q Huang Y Zheng J Guo S He L. Formononetin ameliorates airway inflammation by suppressing ESR1/NLRP3/caspase-1 signaling in asthma. Biomed Pharmacother. (2023) 168:115799. 10.1016/j.biopha.2023.115799 37922653

[45] Zhou Z Zhang P. Formononetin ameliorates the LPS-induced inflammatory response and apoptosis of neuronal cells via NF-*κ*B/NLRP3 signaling pathway. Funct Integr Genomics. (2023) 23:321. 10.1007/s10142-023-01247-1 37847432

[46] Tsujimoto K Jo T Nagira D Konaka H Park JH Yoshimura SI. The lysosomal ragulator complex activates NLRP3 inflammasome *in vivo* via HDAC6. EMBO J. (2023) 42:e111389. 10.15252/embj.2022111389 36444797 PMC9811619

[47] Huang Z Liu Y Huang X. Formononetin may protect aged hearts from ischemia/reperfusion damage by enhancing autophagic degradation. Mol Med Rep. (2018) 18:4821–30. 10.3892/mmr.2018.9544 30320398 PMC6236296

[48] Wang Y Zhao H Li X Wang Q Yan M Zhang H. Formononetin alleviates hepatic steatosis by facilitating TFEB-mediated lysosome biogenesis and lipophagy. J Nutr Biochem. (2019) 73:108214. 10.1016/j.jnutbio.2019.07.005 31520816

[49] Abbastabar M Kheyrollah M Azizian K Bagherlou N Tehrani SS Maniati M. Multiple functions of p27 in cell cycle, apoptosis, epigenetic modification and transcriptional regulation for the control of cell growth: a double-edged sword protein. DNA Repair (Amst). (2018) 69:63–72. 10.1016/j.dnarep.2018.07.008 30075372

[50] Pang X Guan Q Lin X Chang N. Knockdown of HDAC6 alleviates ventricular remodeling in experimental dilated cardiomyopathy via inhibition of NLRP3 inflammasome activation and promotion of cardiomyocyte autophagy. Cell Biol Toxicol. (2023) 39:2365–79. 10.1007/s10565-022-09727-z 35764897

[51] Wu W Fu J Gu Y Wei Y Ma P Wu J. JAK2/STAT3 Regulates estrogen-related senescence of bone marrow stem cells. J Endocrinol. (2020) 245:141–53. 10.1530/JOE-19-0518 32045363

[52] Ding M Bao Y Liang H Zhang X Li B Yang R. Potential mechanisms of formononetin against inflammation and oxidative stress: a review. Front Pharmacol. (2024) 15:1368765. 10.3389/fphar.2024.1368765 38799172 PMC11116718

[53] Diksha Singh L Bhatia D. Mechanistic interplay of different mediators involved in mediating the anti-depressant effect of isoflavones. Metab Brain Dis. (2024) 39:199–215. 10.1007/s11011-023-01302-7 37855935

[54] Zhang C Zhu L Lu S Li M Bai M Li Y. The antidepressant-like effect of formononetin on chronic corticosterone-treated mice. Brain Res. (2022) 1783:147844. 10.1016/j.brainres.2022.147844 35218705

[55] Liu X Wei F Liu H Zhao S Du G Qin X. Integrating hippocampal metabolomics and network pharmacology deciphers the antidepressant mechanisms of Xiaoyaosan. J Ethnopharmacol. (2021) 268:113549. 10.1016/j.jep.2020.113549 33152435

[56] Zhang C Hu L Xie Y Wen J Chen Y. Formononetin improves cognitive behavior in aging rats with chronic unpredictable mild in hippocampal tissue stress by blocking the NF-*κ*B pathway and inhibiting the release of inflammatory factors. Xi Bao Yu Fen Zi Mian Yi Xue Za Zhi. (2023) 39:610–6. 37403720

